# Synthetic Derivates of Progesterone Ameliorate Scopolamine-Induced Cognitive Deficits in Animal Models: Antioxidant, Enzyme Inhibitory, Molecular Docking and Behavioral Correlates

**DOI:** 10.2174/011570159X357722250212094900

**Published:** 2025-03-27

**Authors:** Asif Nawaz, Abdul Sadiq, Nasreena Bashir, Umer Rashid, Farhat Ullah, Shahbaz Khan, Farman Ullah, Mohammad Inam Khan, Muhammad Ayaz

**Affiliations:** 1 Department of Pharmacy, Faculty of Biological Sciences, University of Malakand, Chakdara, 18800 Dir (L), Khyber Pakhtunkhwa, Pakistan;; 2 Department of Clinical Laboratory Sciences, College of Applied Medical Sciences, King Khalid University, Abha, 61421, Saudi Arabia;; 3 Department of Chemistry, COMSATS University Islamabad, 22060 Abbottabad, Pakistan;; 4 Khyber Medical College, Khyber Medical University, Peshawar, Pakistan;; 5 Department of Pharmacy, Kohat University of Science and Technology (KUST), Khyber Pakhtunkhwa (KP), Pakistan;; 6 Public Health Department, College of Health Sciences, Saudi Electronic University, Abha Male 61421, Saudi Arabia

**Keywords:** Neuroprotection, Alzheimer’s disease, steroids, progesterone, cholinesterase’s, antioxidants

## Abstract

**Background:**

Alzheimer's disease (AD) is a neurological disorder characterized by cognitive decline and behavioral distrubance which are expected to significantly affect the patient's quality of life. Previous studies revealed the neuroprotective effects of progesterone. Furthermore, the aim of this study was to assess the neuroprotective potentials of new derivatives of progesterone (AN-1 to AN-5).

**Methods:**

Following compound synthesis and structure elucidation, in vitro antioxidant (DPPH), acetylcholinesterase (AChE) and butyrylcholinesterase (BChE) inhibitory activities, as well as molecular docking studies, were performed, according to the standard procedures and the most potent compound was then subjected to more detailed behavioral studies, including the Y-Maze, Elevated Plus Maze (EPM), and open field tests in scopolamine-induced amnesic animals.

**Results:**

In the DPPH assay, the AN-1 compound at 1000 µg/ml concentration exhibited 83.37 ± 2.03% inhibition of DPPH free radicals with an IC_50_ value of 57.21 µM. Likewise, the compound AN-1 demonstrated 88.94 ± 1.20% inhibition against AChE and 86.78 ± 1.24% inhibition against BChE enzymes at 1000 µg/ml with IC_50_ values of 56.52 and 43.33 µM, correspondingly. In behavioral studies, compound AN-1 demonstrated a significant decline in cognitive impairments and improved working memory as well as locomotor activities of the amnesic animals. Molecular docking studies also demonstrated that the compound AN-1 has promising inhibitory potentials against AChE and BChE enzymes by binding to their active site amino acid residues. The binding energies of AN-1 with both enzymes were -7.6 Kcal/mol for AChE and -8.1 Kcal/mol for BChE.

**Conclusion:**

Based on our findings, it is concluded that the derivatives of progesterone exhibit neuroprotective potential, and further research is needed to extend their neuroprotective role in the treatment of AD.

## 1 INTRODUCTION

Alzheimer's disease (AD) is a highly prevalent neurological disorder characterized by cognitive dysfunctions, behavioral turbulence, and imperfection in routine activities, all of which adversely affect the quality of life for a large number of people [1]. In the United States, AD is among the top five causes of mortality, affecting 5.3 million people [2]. The International Alzheimer’s Association reported about fifty million dementia patients in 2018, and this number will increase to 152 million by the year 2050 [3]. The AD symptoms were reported for the first time by Alois Alzheimer, a German neuropathologist, in 1906. The disease was anticipated as a continuous decrease in cognition and personality alterations due to senile plaques (SPs) and accumulation of neurofibrillary tangles (NFTs) in the brain [4]. In AD patients, a progressive impairment in the neurons of the amygdala and hippocampal area of the brain has been observed [5]. Pathologically, AD is classified as Familial Alzheimer’s disease (FAD) or pre-senile AD and Sporadic Alzheimer’s disease (SAD) or senile dementia. FAD occurs at the age of less than sixty-five years, whereas SAD occurs after 65 years of age [6]. In the AD patients' brains, there is an accumulation of highly phosphorylated tau protein, beta-amyloid plaques (Aβ), and neurofibrillary tangles (NFTs). Moreover, there is a gradual decline in acetylcholine (ACh) and butyrylcholine (BCh) resulting from the deterioration of cholinergic neurons due to the accumulation of these toxic substances and excessive liberation of free radicals [7-10].

One of the treatment strategies for AD involves the use of anticholinergic drugs, which exhibit their therapeutic effect by inhibiting the key enzymes BChE/AChE, responsible for the metabolic degradation of ACh [11]. Likewise, reactive oxygen species (ROS) cause deterioration of lipids, proteins, and nucleic acid and thus result in several neurological disorders [12]. Hence, the antioxidant strategy may be effective for preventing and treating neurological diseases, including AD [13]. Other therapeutic options include inhibitors of monoamine oxidase (MAO) and beta-amyloid cleaving enzyme (BACE1) [8, 14]. Particularly, MAO-B is known to be implicated in the pathophysiology of AD, whereas BACE1 plays a role in the production of Aβ from amyloid precursor protein (APP). Currently, a few AChE inhibitors and noncompetitive N-methyl D-aspartate receptor antagonists (NMDA), called memantine, have been approved by the FDA for the management of AD [15]. Galantamine, donepezil, rivastigmine, and tacrine are the most renowned inhibitors of cholinesterase enzymes for treating AD patients having mild to moderate AD [16]. These drugs only provide symptomatic relief [17]. Apart from this, no anti-amyloid agent is clinically approved, though several compounds are presently under investigation in clinical trials for AD treatment.

**Fig. (1):**
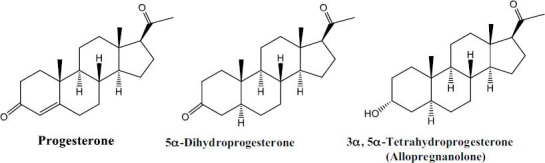
Structures of progesterone and pregnanolone.

Neuronal functions are regulated by neuroactive steroids, which may include exogenous steroids like synthetic steroidal compounds or endogenous hormonal steroids and neuro-steroids [18]. Progesterone and pregnenolone are common neuro-steroids (Fig. **1**), which are reported for their significance in nervous system maturation and prevention of ischemia and neuronal toxicity [19]. Progesterone is an important neuro-steroid and an endogenous neuromodulator, formed by the central nervous system (CNS) and mainly concerned with various pathological and physiological activities of CNS [20]. A number of studies have reported the neuroprotective potential of progesterone and demonstrated these hormones offer protection against neurodegenerative disorders like traumatic brain injury, stroke, and AD [21]. Progesterone is converted to neuroactive metabolites, including 5α-dihydro-progesterone and 3α,5α-tetrahydro-progesterone, which contributes to its neuroprotective potentials [22]. The 3α, 5α-tetrahydro-progesterone is reported to reverse cognitive deficits in AD animal models [23]. Likewise, progesterone derivatives, including dihydro-progesterone (DHP) and tetrahydro-progesterone (THP), exhibit neuroprotective actions that have been reported in streptozotocin-induced diabetic neuropathy and nerve crush experimental models [24, 25]. The cholinergic hypothesis suggests that impairment or loss of memory among AD patients is due to a lack of cholinergic functions in the synaptic region of the brain [26]. The role of anticholinesterase enzymes is to increase the level of acetylcholine in the brain, making them a vital target for drug design against AD [27]. The cholinesterase inhibitory potential of nitrogenous derivatives of progesterone has been reported previously [28]. The synthesized nitrogenous progesterone derivatives have demonstrated considerable inhibition against AChE/BChE enzymes [28]. Considering the neuroprotective potential of progesterone as well as its derivatives, this study aimed to prepare new derivatives of progesterone (AN1-5) and to determine their anti-amnesic and neuroprotective effects using *in-vitro*, *in-silico*, and *in-vivo* models of AD.

## 2 MATERIALS AND METHODS

### 2.1 General

All the chemicals, solvents, and reagents were obtained from authorized commercial vendors. Galantamine HBr, progesterone, 4-Methoxybenzylamine, 4-Methylbenzylamine, 3,4-Dimethoxybenzylamine, and 4-Chlorobenzylamine were obtained from Sigma-Aldrich, whereas scopolamine was purchased from Shanghai Macklin, China. Burker spectrometer was used to record NMR spectra of ^13^C at 75/100 MHz and ^1^H at 300/400 MHz. Plates of thin layer chromatography (TLC) prior coated with silica gel having 0.25 mm layer thickness (Merck) were used to monitor the progress of reactions.

### 2.2 General Synthesis of Progesterone Derivatives

Progesterone derivatives synthesis was carried out by reacting commercially available amine with progesterone. A small amount of acetic acid in ethanol was added to catalyse the reaction. To monitor the progress of reactions, TLC was utilized, and after 4-8 hours, the reaction products were precipitated out at completion. The ^1^H and ^13^C NMR analyses were used to confirm the structure of synthesized compounds [29].

#### 2.2.1 Synthesis of Compound AN1-AN5

Compounds AN-1, AN-2, AN-3, AN-4, and AN-5 were synthesized by reacting progesterone (314.46 mg, 1 equivalent, 1 mmol) with 4-Methoxybenzylamine (137.18 mg, 1 equivalent, 1 mmol), 4-Methylbenzylamine (121.18 mg, 1 equivalent, 1 mmol), 3,4-Dimethoxybenzylamine (167.21 mg, 1 equivalent, 1 mmol), 4-Chlorobenzylamine (141.60 mg, 1 equivalent, 1 mmol) and benzylamine (107.15 mg, 1 equivalent, 1 mmol) respectively. A catalytic amount of acetic acid (11.45 µl, 20 mol %) in ethanol (1 ml, 1M) was used to catalyse the reaction. The reactions were completed in 4-8 hours.

### 2.3 Cholinesterase Inhibition Assays

The cholinesterase inhibitory potentials of our synthesized compounds were evaluated against cholinesterase enzymes (AChE/BChE). The AChE (source *Electric eel*) and BChE (source equine serum) were used in the study following previously published Ellman's assay [1, 30, 31]. The assay basic principle involves the hydrolytic breakdown of acetylthiocholine iodide (ATChI) by AChE and butyryl thiocholine iodide (BTChI) by BChE enzymes, leading to the formation of an intermediate compound which is subsequently complexed with 5,5-dithio bis nitrobenzoic acid (DTNB) to form a UV-detectable compound. The yellowish color product indicates the hydrolysis of ATChI and BTChI substrates by respective enzymes, which were analyzed at 412 nm using a UV-visible spectrophotometer. Methanolic solutions of standard drug galantamine (positive control) and test samples were prepared in various concentrations ranging from 62.25 to 1000 µg/ml. A test sample of 1000 µl was taken in a cuvette, to which enzyme solution (50 µl) and DTNB reagent of 50 µL were added. The AChE (0.03 U/ml) and BChE (0.01 U/ml) solutions were prepared in 0.1 M phosphate buffer having pH 8.0. The ATChI and BTChI substrate solutions (0.0005 M) and 0.0002273 M solution of DTNB were prepared using distilled water and stored at 8°C before further use. For 15 minutes, the solution mixture was incubated at 30ºC, after which a solution of the substrate (50 µl) was added and then subjected to spectroscopic analysis. Using a double beam spectrophotometer, the sample absorbance was measured for 4 minutes at 412 nm beside the time of reaction. The positive control or standard cholinesterase inhibitor was galantamine, while the sample of negative control had all the reaction components without the test sample. All values were taken in triplicate under the same conditions. The enzyme (AChE/BChE) inhibition by our test compounds and standard drug galantamine and enzyme activity were determined by the given formulas;

V = 
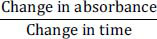


Enzymatic activity percentage=V/V_max_x100

Percent inhibition of enzyme = 100 – enzymatic activity percentage

V is the reaction rate in the inhibitor's presence, whereas V_max_ is the reaction rate in the absence of the inhibitor.

### 2.4 DPPH Antioxidant Assay

To evaluate the *in-vitro* antioxidant potential of the synthesized derivatives, a previously reported 1,1 Diphenyl, 2 Picrylhydrazyl (DPPH) assay was utilized [32]. The DPPH solution was prepared by dissolving twenty-four mg DPPH in methanol (100 ml). Test sample solutions in various concentrations (62.50 to 1000 μg/mL) were also prepared. From each dilution, 1 ml sample solution was taken to which 1 ml solution of DPPH was added. The solution mixture was incubated at 23°C for 30 minutes in the dark and then subjected to a UV-visible spectrophotometer to note the absorbance at 517 nm. The absorbance values of all samples were taken in triplicate. Ascorbic acid served as positive control, while negative control only contained a solution of DPPH having no test samples. The % free radical scavenging potential of our compounds was determined by the given formula.

% free radical scavenging activity = 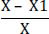
x100

X is the control absorbance, whereas X1 is the absorbance of the test sample.

### 2.5 *In-vivo* Studies

#### 2.5.1 Experimental Animals

In our study, experimental animals (albino mice male/female weighing 18-32 gm) were obtained from the National Institute of Health (NIH), Islamabad, Pakistan. These animals were provided adequate food, water, and light (*ad libitum*). Appropriate conditions were maintained as per laboratory specifications, including a temperature of 22°C a dark and light cycle of 12 hours each [33]. As per the Animals Scientific Procedure Act 1986, UK, the *in-vivo* studies were performed on experimental animals from 9:00 am to 4:00 pm [34]. The departmental committee of research ethics of the Pharmacy Department, University of Malakand, approved the study protocol *via* reference no DREC/20210622/50.

#### 2.5.2 Acute Toxicity Studies

Acute toxicity was carried out for the safety profile assessment of our test compounds. For this purpose, the mice were grouped into six different groups (n = 6). The test compound was given to each mouse at different doses (25 to 1000 mg/kg) *via* the intraperitoneal (i.p) route. Changes in behaviour such as convulsions, hyperactivity, writhing, sedation, righting reflex, aggressiveness, grooming, ataxia, motor activity (increased or decreased), respiratory arrest, and mortality were observed after the administration of sample solution for the first two hours and then again for 24 hours [35].

#### 2.5.3 Grouping of Animals

The mice were divided into the following five groups, and each group had six animals. The standard drug and test samples were carefully administered to the mice *via* the intraperitoneal route.

Group I (Saline group) = The normal control group was only given normal saline.

Group II (Disease group) = The disease group only received 1 mg/kg scopolamine.

Group III (Galantamine treated group) = Animals received 8 mg/kg galantamine and scopolamine 1 mg/kg.

Group IV (Progesterone treated group) = Animals treated with 10 mg/kg progesterone and scopolamine 1 mg/kg.

Group V (Test compound group) = Animals received 10 mg/kg progesterone derivative (AN-1) and 1 mg/kg scopolamine.

### 2.6 Behavioral Studies

#### 2.6.1 Y Maze Test

To determine the effect of synthesized compounds on short-term learning and memory of experimental mice, a Y-Maze test was used. The Y-Maze apparatus consists of three arms that are fixed at 120° angle from each other and have 30 cm length, 20 cm height, and 8 cm width dimensions [36]. After 30 minutes of giving test compounds intraperitoneally, the mouse was positioned at the arm end in such a way that it faced away from the midpoint of the apparatus and was permitted to search the apparatus for five minutes freely. The arm entry sequence and number by each mouse were recorded for 5 minutes using a digital camera. Alternation is defined as the successive entry into 3 dissimilar arms by a mouse (*i.e*., CBA, BCA, ABC). When the back paws of a mouse were found inside an arm wholly, the arm entry was considered completed. Seventy percent v/v ethanol was used for cleaning the apparatus and to avoid smell cues. The spontaneous alternation performance (SAP) percentage was calculated using the following formula [37]:

% alternation = [(alternations number) / (total arm entries number – 2)] × 100

#### 2.6.2 Elevated Plus Maze (EPM)

The EPM behavioral test was performed to assess the learning abilities and long-term spatial memory of the experimental animals [38]. The EPM apparatus has the appearance of a plus sign which was 50 cm elevated from the ground. The apparatus has two open arms (50 cm long and 10 cm wide), and two closed arms having dimensions of 20, 50, and 10 cm in height, length, and width, respectively. The open and closed arms were joined with a 10 × 10 central square [38].

##### 2.6.2.1 Training Trials

After 30 minutes of the intraperitoneal drug administration on the seventh day of treatment, mice of each group were placed over the open arm end of the EPM apparatus in such a way that their faces were away from the midpoint of the apparatus. Transfer latency (TL) is the time a mouse takes to enter the closed arm entirely with all its legs from the open arm of the apparatus. The time of initial transfer latency (ITL) was observed, and then the animals were allowed freely for 1.5 minutes to search the EPM apparatus.

##### 2.6.2.2 Test Trials

After twenty-four hours of the training period, test trials were conducted to determine the retention of tasks that were learnt during training. Retention transfer latency (RTL) was measured by placing the experimental mice at the open end of the apparatus, ensuring that they faced away from its midpoint. The mouse was allowed to enter the closed arm entirely with all its legs. A decline in TL compared to ITL indicated an improvement in memory.

#### 2.6.3 Open Field Test

Open field behavioral test was carried out for the assessment of locomotor activities like the number of line crossing [39]. The apparatus was made from plywood having 81×81×28.5 cm dimensions. Sixteen squares (20×20 cm) were made on the base of the apparatus by drawing four horizontal and four vertical lines. The total number of line crossings was observed for five minutes with the help of a digital camera by positioning each mouse at the apparatus midpoint and permitted to explore the apparatus openly. Furthermore, exploration time spent by mice in central and peripheral areas of the apparatus was also recorded by allowing the mice for 30 minutes to explore the apparatus [40].

### 2.7 Molecular Docking Studies

Induced fit (flexible) molecular docking was also carried out using Molecular Operating Environment (MOE) software to determine the binding behavior of AN1 progesterone derivative in the binding sites of AChE (PDB ID = 1EVE) and BChE (PDB ID = 4BDS). The water of crystallization in the binding site of AChE was retained. The preparation of ligands, enzymes and docking studies were carried out by using our previously reported protocols. Discovery Studio Visualizer (DSV) was used to visualize the 3D and 2D interaction plots [41].

### 2.8 Statistical Analysis

Graph prism 8.0.1 version was utilized for statistical analysis. To each data set, ANOVA was applied persuaded by appropriate *post hoc* tests for data significance. The *p*-value ˂ 0.05 was deemed as significant statistically.

## 3 RESULTS

### 3.1 Synthesis of Progesterone Derivatives

We prepared five new derivatives of progesterone (AN1- AN5), whose structures are shown in Fig. (**2**), and details of the selected derivatives are provided below. The reaction time for all of our compounds was 4-8 hours.

### 3.2 AN1 Compound Synthesis

Chemically compound AN1 is (8S,9S,10R,13S,14S)-17-((Z)-1-((4-methoxybenzyl) imino) ethyl)-10,13-dimethyl-1,2,6,7,8,9,10,11,12,13,14,15,16,17-tetradecahydro-3H-cyc-lopenta[a]phenanthren-3-one. The percent yield of AN1 was 87% with Rf value 0.32 using n-hexane and ethyl acetate solvent system in a ratio of 70:30. The ^1^H NMR (chloroform D, 400 MHz): 0.67 (s, 3H), 0.95-1.16 (m, 2H), 1.18 (m, 3H), 1.22-1.32 (m, 1H), 1.40-1.54 (m, 2H), 1.61-1.75 (m, 5H), 1.83-1.89 (m, 1H), 2.01-2.07 (m, 2H), 2.12 (s, 3H), 2.13-2.16 (m, 1H), 2.17-2.47 (m, 5H), 2.53 (t, *J* = 9.05 Hz, 1H), 3.84 (s, 3H), 4.56 (s, 2H), 5.76 (s, 1H), 6.87 (d, *J* = 8.58 Hz, 2H) and 7.12 (d, *J* = 8.61 Hz, 2H). The ^13^C NMR (chloroform D, 100 MHz): 14.87, 16.61, 18.18, 20.22, 21.48, 21.78, 23.78, 25.22, 26.42, 27.93, 28.28, 28.83, 31.30, 33.35, 35.39, 36.78, 42.45, 43.59, 51.60, 56.95, 126.59, 127.97, 128.75, 132.50, 155.35, 171.25, 173.08 and 201.00.

### 3.3 AN2 Compound Synthesis

Chemically AN2 compound is (8S,9S,10R,13S,14S)-17-((Z)-1-((4-methylbenzyl) imino) ethyl)-10,13-dimethyl-1,2,6,7,8,9,10,11,12,13,14,15,16,17-tetradecahydro-3H-cyc-lopenta[a]phenanthren-3-one. The percent yield if this compound was 84% with Rf value of 0.38 using n-hexane and ethyl acetate solvent system in a ratio of 70:30.The ^1^H NMR (chloroform D, 400 MHz): 0.81 (s, 3H), 0.97-1.13 (m, 2H), 1.18 (m, 3H), 1.23-1.75 (m, 9H), 1.84-1.89 (m, 1H), 2.02-2.07 (m, 2H), 2.12 (s, 3H), 2.23 (s, 3H), 2.30-2.46 (m, 5H), 2.54 (t, *J* = 8.74 Hz, 1H), 4.54 (s, 2H), 5.73 (s, 1H), 6.98 (d, *J* = 8.50 Hz, 2H) and 7.10 (d, *J* = 8.51 Hz, 2H). The ^13^C NMR (chloroform D, 100 MHz): 13.67, 16.09, 17.38, 21.47, 22.45, 22.93, 23.67, 25.26, 26.31, 28.81, 29.38, 31.31, 32.03, 32.67, 34.86, 35.81, 42.43, 44.20, 52.81, 126.70, 128.07, 129.20, 131.93, 156.26, 168.12, 170.04 and 202.20.

### 3.4 AN3 Compound Synthesis

Chemically compound AN3 is (8S,9S,10R,13S,14S,17S)-17-((Z)-1-((3,4-dimethoxybenzyl)imino)ethyl)-10,13-dimethyl- 1,2,6,7,8,9,10,11,12,13,14,15,16,17-tetradecahydro-3H-cyclo-penta[a]phenanthren-3-one. Further, the percent yield of AN3 was 74% with Rf value 0.24 using n-hexane and ethyl acetate solvent system in a ratio of 70:30. The ^1^H NMR (chloroform D, 400 MHz): 0.66 (s, 3H), 0.93-1.14 (m, 2H), 1.18-1.24 (m, 3H), 1.25-1.35 (m, 1H), 1.39-1.47 (m, 2H), 1.60-1.74 (m, 5H), 1.81-1.86 (m, 1H), 1.99-2.05 (m, 2H), 2.09 (s, 3H), 2.11-2.14 (m, 1H), 2.17-2.41 (m, 5H), 2.49 (t, *J* = 8.77 Hz, 1H), 3.81 (s, 3H), 3.87 (s, 3H), 4.55 (s, 2H), 5.74 (s, 1H), 6.89 (s, 1H), 7.01 (d, *J* = 7.68 Hz, 1H) and 7.11 (d, J = 7.70 Hz, 1H). The ^13^C NMR (chloroform D, 100 MHz): 15.32, 16.75, 18.33, 21.01, 21.64, 21.72, 24.30, 25.96, 27.06, 27.64, 27.95, 28.32, 31.75, 34.65, 37.32, 37.85, 44.05, 47.50, 53.74, 55.37, 57.68, 119.64, 125.37, 126.25, 129.96, 152.46, 170.55, 172.43 and 204.42.

### 3.5 AN4 Compound Synthesis

Chemically AN4 compound is (8S,9S,10R,13S,14S,17S)-17-((Z)-1-((4-chlorobenzyl)imino)ethyl)-10,13-dimethyl-1,2, 6,7,8,9,10,11,12,13,14,15,16,17-tetradecahydro-3H-cyclopenta [a]phenanthren-3-one. The percent yield of AN4 was 71% with Rf value of 0.28 using n-hexane and ethyl acetate solvent system in the ratio of 70:30. The ^1^H NMR (chloroform D, 400 MHz): 0.71 (s, 3H), 1.05-1.14 (m, 2H), 1.21-1.26 (m, 2H), 1.28-1.66 (m, 8H), 1.77-1.91 (m, 3H), 1.97-2.04 (m, 2H), 2.06 (s, 3H), 2.20 (s, 3H), 2.35-2.51 (m, 5H), 2.50 (t, *J* = 8.62 Hz, 1H), 4.46 (s, 2H), 5.71 (s, 1H), 6.68 (d, *J* = 7.54 Hz, 2H) and 6.94 (d, *J* = 7.53 Hz, 2H). The ^13^C NMR (chloroform D, 100 MHz): 11.63, 14.63, 17.53, 20.31, 23.23, 23.65, 24.37, 28.76, 28.95, 29.38, 31.67, 32.49, 33.76, 35.31, 35.71, 45.63, 48.52, 50.04, 123.52, 124.45, 128.50, 130.00, 153.46, 167.46, 169.44 and 201.69.

### 3.6 AN5 Compound Synthesis

Chemically AN5 compound is (8S,9S,10R,13S,14S)-17-((Z)-1-(benzylimino)ethyl)-10,13-dimethyl-1,2,6,7,8,9,10,11, 12,13,14,15,16,17-tetradecahydro-3H cyclopenta[a]phenan-thren-3-one. The percent yield of AN5 was 92% with 0.42 Rf value using n-hexane and ethyl acetate solvent system in a ratio of 70:30. The ^1^H NMR (chloroform D, 400 MHz): 0.72 (s, 3H), 1.01 (s, 3H), 1.03-1.17 (m, 1H), 1.23-1.38 (m, 2H), 1.42-1.64 (m, 9H), 1.68-1.78 (m, 2H), 1.81-1.88 (m, 2H), 1.92 (s, 3H), 1.94-2.06 (m, 2H), 2.21-2.41 (m, 3H), 2.84 (t, *J* = 8.91 Hz, 1H), 5.38 (bs, 1H), 7.33-7.40 (m, 2H) and 7.46-7.54 (m, 3H). The ^13^C NMR (chloroform D, 100 MHz): 14.36, 16.75, 18.43, 21.20, 25.80, 27.73, 29.48, 30.29, 31.50, 33.50, 34.43, 35.23, 36.73, 40.20, 42.60, 43.18, 50.44, 52.19, 56.78, 128.08, 128.18, 128.72, 128.79, 135.87, 170.97, 171.97 and 202.85.

### 3.7 *In-vitro* Inhibition of Acetylcholinesterase and Butyrylcholinesterase

The results of AChE/BChE inhibitory potentials of the synthesized compounds (**AN-1** to **AN-5**) are summarized in Table **1**. In the AChE inhibition assay, AN1 was considerably active against the enzyme with 88.94 ± 1.2% inhibition (1000 µg/ml) with an IC_50_ of 56.52 µM. Compounds AN3 & AN5 demonstrated IC_50_ values of 133.18 and 156.44 µM persuaded by AN2 and AN4 compounds with 173.16 and 213.45 µM IC_50_ values, correspondingly. Similarly, compound AN1 showed the highest BChE inhibition (86.78 ± 1.24%) at 1000 µg/ml concentration with IC_50_ of 43.33 µM. The IC_50_ values of 63.007 and 111.54 µM were observed for compounds AN-5 and AN-3, followed by compounds AN-2 and AN-4 with 138.04 and 148.95 µM IC_50_ values, respectively, against BChE. The standard drug galantamine demonstrated 91.88 ± 2.45% inhibition against AChE and 89.49 ± 1.93% inhibition against BChE with IC_50_ of 53.83and 27.31 µM correspondingly at 1000 µg/ml concentration.

### 3.8 DPPH Assay

A concentration-dependent antioxidant activity against DPPH free radicals was observed for the synthesized compounds (**AN-1** to **AN-5**), as shown in Table **2**. Compounds AN-1, AN-2, AN-3, AN-4, and AN-5 exhibited 83.37 ± 2.03%, 71.47 ± 1.83%, 72.32 ± 1.37%, 69.87 ± 2.81% and 73.12 ± 1.04% inhibition at the highest tested concentration (1000 µg/ml). The IC_50_ values for these compounds were 57.21, 62.47, 86.37, 188.20, and 110.45 µM respectively. The positive control (ascorbic acid) showed 91.17 ± 1.62% inhibition against DPPH free radicals at a concentration of 1000 µg/ml with IC_50_ of 54.05 µM.

### 3.9 Acute Toxicity Study Results

On the basis of acute toxicity results, the doses of our synthesized compounds (AN-1) was decided for *in-vivo* studies. The toxic effects of the test compound were evaluated in six different groups (n = 6) of mice at different doses ranging from 25 to 1000 mg per kg body weight. Our test compounds up to a dose of 500 mg/kg did not show any toxic effects and were well tolerated by mice. Up to this dose, no mortality or unusual behavioral response was observed. Whereas, at 1000 mg/kg dose, mortality was observed. Hence, up to a dose of 500 mg/kg, our compound was safe and was toxic at 1000 or more than 1000 mg/kg dose.

### 3.10 Results of the Behavioral Studies

All of the synthesized compounds showed anti-Alzheimer potential in *in-vitro* studies. Among the compounds, AN1 was most potent so was subjected to further *in-vivo* behavioral studies including EPM, Y-Maze and open field tests.

#### 3.10.1 EPM Test Results

The transfer latency (TL) time was noted in the EPM test for the assessment of beneficial neurocognitive effects of AN1. Fig. (**3**) shows the initial transfer latency ITL time (day 1), whereas Fig. (**4**) shows retention transfer latency (RTL) on day 2, which indicates the learning memory and tasks. The decline in the RTL time suggests improvement in memory. Values of ITL (73 sec) and RTL (67 sec) of the untreated scopolamine-induced amnesic group (disease group) were considerably different from the control group (ITL 38.33 sec) and (RTL 25.0 Sec) and with a statistically significant (*p* less than 0.001). The AN1-treated amnesic animals (ITL 50.16 sec) and (RTL 45.33 sec) were comparable with the standard galantamine-treated amnesic group (ITL 47.33 sec) and (RTL 38.33 sec) and demonstrated a significant (*p*<0.01) decline in ITL as compared to disease group indicating improvement in memory. The progesterone group also demonstrated a considerable decline in time of ITL (56.5 sec) and the RTL was 50.33 sec.

#### 3.10.2 Y-maze Test Results

Assessment of rodent’s spontaneous alternation (percent) behavior is an important parameter considered in the Y-maze test to evaluate retention of spatial or short-term memory. The results of the Y-maze test are shown in Fig. (**5**). A considerably low (*p*<0.001) spontaneous alternation was observed for the untreated scopolamine-induced amnesic group (22.27%) when compared with the saline group. The results of other treated groups were compared to the untreated scopolamine-induced amnesia (disease) group. Considerable improvement in spontaneous alternation behavior (42.72%) was noted for the AN-1 treated group (test group) in comparison to untreated amnesic animals (22.27%). The spontaneous alternation of amnesic animals treated with progesterone was (40.001%) which is of considerable importance and comparable with the AN-1 treated group, yet the percent alternation was not significantly different. The galantamine-treated amnesic animals demonstrated improved spontaneous alternation behavior (47.56%) and were significantly (*p*<0.01) different from the untreated disease group.

#### 3.10.3 Open Field Test (OFT) Results

**Fig. (6):**
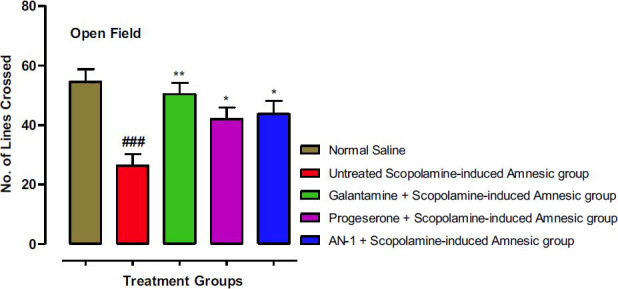
Results of different groups of animals in OFT on the number of lines crossing. One-way ANOVA followed by Dunnett’s test was applied to the data. The untreated scopolamine-induced amnesia group (Disease group) was compared to the control group (saline-treated normal animals’ group), whereas the other test groups were compared to the disease group. Symbols * and ** represent *p* values less than 0.05 and 0.01 correspondingly. The value of the scopolamine group was indicated by symbol ### (*p*<0.001).

The locomotor activity of experimental animals was assessed by observing the number of lines crossed time spent in central and peripheral regions of the OFT apparatus, and results are given in Figs. (**6**, **7** and **8**) correspondingly. The number of lines crossed (26.33) by the scopolamine-induced amnesic group (disease group) animals was significantly (*p*<0.001) less than the saline control group (54.5). Galantamine-treated amnesic animals showed a significant (*p*<0.01) increase in the number of lines crossed (50.33), whereas progesterone (42) and AN1-treated amnesic animals (43.66) demonstrated significant (*p*<0.05) rise in number of lines crossed when compared with scopolamine-induced amnesic group (26.33), indicating improvement in the explorative behavior of amnesic animals (Fig. **6**).

Yet from another perspective, the time spent by animals in the central compartment of the apparatus was assessed as more anxious animals spent less time in the central area. As shown in Fig. (**7**), amnesic animals treated with standard galantamine spent more time in the central area (13 mint), which was significantly (*p*<0.05) more than untreated amnesic animals (4.66 Mint). Among other groups, progesterone-treated animals, AN-1-treated animals, and normal animals spent 9.66, 10.33 and 10. 0 mint respectively in the central area.

A significantly high (*p*<0.001) time was spent by the scopolamine/disease group in the peripheral area in comparison to the normal saline group. The AN1 test compound and standard control galantamine groups displayed significantly (*p*<0.01) less time in the peripheral area. A significant (*p*˂0.05) decline in time spent peripherally was also displayed by the progesterone group, as shown in Fig. (**8**).

### 3.11 Docking Studies

Induced fit (flexible) molecular docking was also carried out using Molecular Operating Environment (MOE) software to determine the binding behavior of AN1 progesterone derivative in the binding sites of AChE (PDB ID = 1EVE) and BChE (PDB ID = 4BDS). The interaction plots are shown in Figs. (**9** and **10**), respectively.

**Fig. (9):**
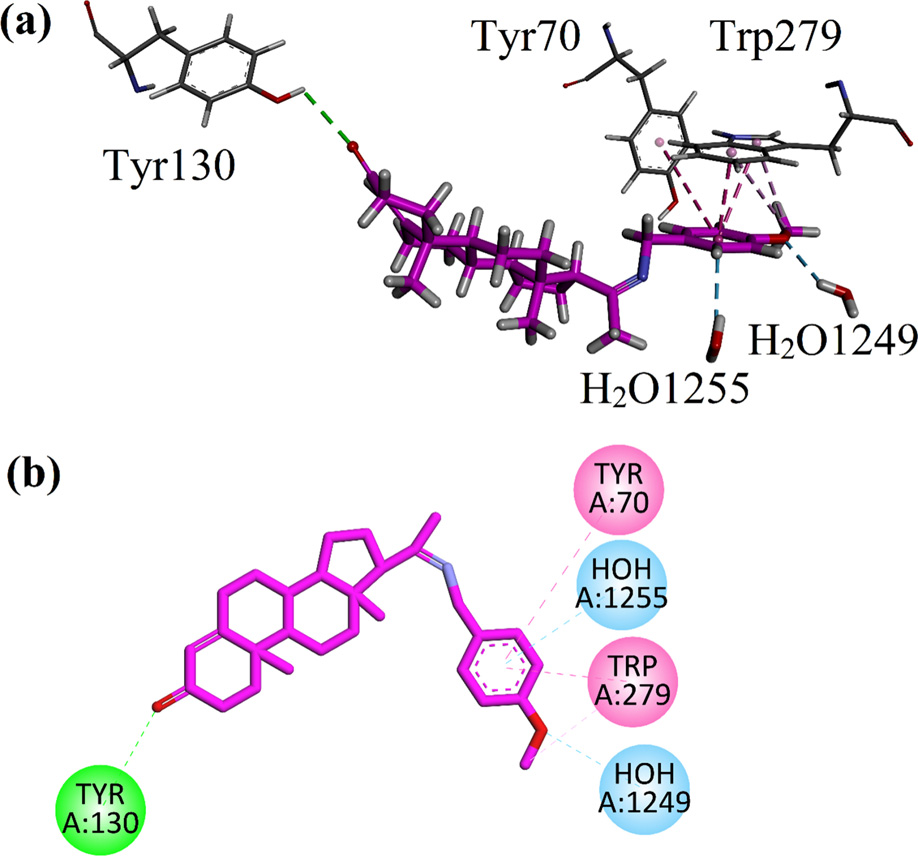
(**a**) 3D (**b**) 2D visualization of the interaction of AN1 compound in the binding site of AChE (PDB ID = 1EVE) after induced fit docking.

In the flexible docking (induced fit), water molecules present in the binding site of *Tc*AChE were not removed. These water molecules interact with HOH1249 and 1255. While the 4-methoxyphenyl ring interacts with important residues (Tyr70 and Tyr279) present in the peripheral anionic site (PAS) of the target *via* π-π interactions. A hydrogen bond with Tyr130 was also observed (Fig. **9**). The computed binding energy value for AN1 in the binding site of AChE is -7.6 Kcal/mol.

The binding site of BChE (PDB ID = 4BDS) contains no water molecules. The computed binding energy value for AN1 in the binding site of BChE was -8.1 Kcal/mol. The phenyl ring forms π-π stack interactions with Phe329 and Tyr332. While Gly121 forms conventional hydrogen bond interactions with carbonyl oxygen, Key amino acid residue Trp82 interacts target *via* π-alkyl interactions.

## 4 DISCUSSION

Alzheimer's disease (AD) is a highly complex neurodegenerative disorder linked with progressive cognitive decline and poses a considerable burden on global healthcare, thus needing more effective therapeutic strategies [42]. The most common issues associated with AD include cognitive dysfunction, sleep abnormalities, fluctuations in behavior, and emotional variation [43]. In AD patients, a persistent impairment in the neurons of the amygdala and hippocampal regions of the brain has been observed [5]. Furthermore, aggregation of Aβ plaques, formation of NFTs, highly phosphorylated tau protein, decreased concentration of acetylcholine (ACh) and butyryl choline (BCh), as well as free radicals induced neuronal damages are the major pathophysiological hallmarks of AD [44, 45].

It is well known that cholinergic pathways are linked with the pathophysiology of AD and dementia since these pathways extend to the hippocampus and cerebral cortex [46]. A number of studies on cognitive incapacitations in AD due to cholinergic deficits have been reported [47]. In our study, we used scopolamine to induce amnesia in mice, as it is an antagonist of cholinergic receptors and widely used for inducing memory impairments *via* blockade of central M-1 muscarinic receptors. To evaluate the activity of nootropic agents in memory and learning diseases, the scopolamine induced amnesia animals model is widely used [48]. Cholinergic agents have been reported to restore amnesic effects in experimental animals as well as in humans produced by scopolamine [49]. Cholinesterase (AChE and BChE) enzyme inhibitors prevent the breakdown of ACh, which has a significant role in the regulation of autonomic and somatic nervous systems [50]. Hence, AChE inhibitors prevent the degradation of ACh, resulting in an increased level of ACh and, therefore, reversing the memory or learning impairments induced by scopolamine. Low ACh levels in the neuronal synaptic cleft of AD patients have been reported previously [51]. In the synapse, signal transmission is dependent on ACh, and cholinesterases *via* termination of ACh decrease its actions [52]. Thus, the use of cholinesterase inhibitors is among the most useful strategies for the management of AD [53]. These agents are also useful in other neurological disorders, including Parkinson's disease, ataxia, and dementia [54].

Galantamine, donepezil, tacrine, and rivastigmine are among the clinically approved cholinesterase inhibitors for the management of mild to moderate cases of AD [16]. Among these drugs, galantamine, which was also used as a positive control in our study, is the most noteworthy cholinesterase inhibitor [55]. All of our synthesized progesterone derivatives (**AN-1** to **AN-5**) demonstrated considerable inhibitory potentials against AChE & BChE enzymes in dose dose-dependent manner. Most importantly, the inhibition of compound AN-1 was comparable to that of the standard drug galantamine. Compound AN-1 exhibited 88.94 ± 1.2 and 86.78 ± 1.24% inhibition at 1000 µg/ml concentration against AChE & BChE enzymes with 56.52 and 43.33 µM IC_50_ values correspondingly. The other synthesized progesterone derivatives were also effective against AChE & BChE enzymes and demonstrated anticholinesterase activities in concentration-dependent manner.

An antioxidant (DPPH) assay was also performed to evaluate the antioxidant potential of our synthesized compounds. Antioxidants keep the body healthy by preventing tissues from oxidative damage due to free radicals [56]. Free radicals are implicated in the development of several diseases, including ischemic heart diseases, neurodegenerative diseases, neurological diseases, immune system destruction, cancer, atherosclerosis, reperfusion injury of the tissues, gastritis, arthritis, diabetes, and injury of CNS and other tissues [45]. In AD, the formation of β-amyloid peptides initiates the progression of inflammation, disrupts mitochondrial function, and releases a large number of free radicals, leading to neuronal damage in the brain of AD patients [57]. Antioxidants reduce inflammation by scavenging free radicals, making them useful in the treatment of AD [58]. All of our prepared derivatives (AN1-AN5) demonstrated *in-vitro* antioxidant activity against DPPH free radicals.

Behavioral studies on scopolamine-induced amnesic animals’ were performed using Y-maze, EPM, and open-field tests to evaluate the beneficial effects of synthesized compounds on the cognitive and locomotor abilities of the animals. In the EPM test, the effect of AN1 on the time of transfer latency (initial & retention) was observed for the assessment of cognitive performance. On the 7^th^ day of treatment, ITL time was noted for each mouse, which reflected the learning behavior of the animal. After 24 hours (on the 2^nd^ day), RTL time was observed, which reflected retention of learned tasks. A decrease in RTL time indicated improvement in memory. The RTL time of our test compound AN-1 and standard drug galantamine was significantly less than the latency time of the scopolamine/disease group, which indicates improved cognitive effects of AN1.

The Y-maze model was utilized for the evaluation of short-term memory, learning skills and general motion of the test mice to search and adopt new environments [59]. The apparatus consists of three arms connected at an angle of 120° with each other [36]. Normal mice generally explore new arms instead of recently visited arms, and such behavior is controlled by the hippocampus, prefrontal cortex, septum, and basal forebrain [60]. In the Y-maze test, the percent spontaneous alternation was the parameter used to evaluate the retention of spatial or short-term memory. A rise in % spontaneous alternation behavior was noted for the treatment groups in comparison to the scopolamine/disease group, indicating memory improvement. The AN1 demonstrated 42.72 ± 9.41*% spontaneous alternation in comparison to 22.27 ± 5.39% shown by the scopolamine/disease group.

The open-field test was carried out to determine the exploratory and anxiolytic behavior of the mice [61]. The locomotor performance of the animals was determined by observing the number of lines crossed and time spent in the central as well as in the peripheral area of the box. The anxiolytic effect is indicated by crossing an increased number of lines, spending more time centrally and less time in the peripheral areas of the apparatus. The AN-1 and galantamine showed high anxiolytic and exploratory behavior as compared to scopolamine-induced amnesic (disease group). The major clinical symptom that is linked with neurodegenerative disorders is anxiety [62], and due to the anxiolytic effects of our compound AN-1, it may be beneficial for the management of neurological disorders.

Induced fit (flexible) molecular docking was also carried out using MOE software to determine the binding behavior of progesterone derivative AN1 within the binding site of AChE and BChE [61, 63]. The AChE structure consists of 537 amino acids, which includes 14α helices that basically surround the 12β sheets. BChE has structural similarities to the binding site of AChE. Both AChE and BChE have a Peripheral anionic site, catalytic anionic site, catalytic triad, oxyanion hole, and acyl pocket in their binding site, but the structure of both have key similarities and differences in their structures in terms of amino acids. The AChE esoteric site, also named as catalytic triad, comprised His 440, Glu327, and Ser200, whereas the BChE comprised His338, Glu325, and Ser198 in its catalytic triad. The AChE catalytic site (CAS) comprised of Phe331, Phe330, Tyr130, and Trp84, while in BChE, the Ala328 has replaced the Phe330, and key residue tryptophan is preserved in the binding site as Trp82. The replacement of Phe330 results in a lack of inhibition affinity of BChE as compared to AChE. The Oxyanion hole of AChE consists of Ala201, Gly119, and Gly118, which are also conserved in the BChE as Ala119, Gly117, and Gly116. The acyl pocket shape in AChE is determined by aromatic amino acids Phe290 and Phe288, while in BChE, the substitution of phenylalanine residues with Leu288 and Val286 increases the acyl pocket's size, reducing enzyme specificity. The peripheral anionic site (PAS) of AChE is comprised of amino acids Tyr334, Trp279, Tyr121, Asp72, and Tyr70; it is located near the active gorge of the binding site [64].

In the docking studies, we did not remove the water molecules present in the binding site of *Tc*AChE. Koellner *et al*. presented a detailed discussion on the active-site gorge and Buried Water molecules in crystal structures of AChE from *Torpedo californica* [64, 65]. Studies have shown that acetylcholinesterase interrelates with amyloid beta (Aβ) in the proximity of the peripheral anionic site (PAS) *via* a hydrophobic environment. Hence, small molecules having the ability to bind with peripheral anionic sites will inhibit amyloid beta aggregation and AChE. The synthesized derivative AN1 has shown the ability to bind with PAS residues (Tyr70 and Tyr279), and this may result in inhibiting Aβ aggregation [66]. The compound AN-1 showed a higher binding affinity for targeted cholinesterase enzymes with a binding energy of -7.6 Kcal/mol for AChE, and this energy was observed as-8.1 Kcal/mol for BChE. These binding scores indicated that the compound AN-1 has promising potential to inhibit AChE and BChE enzymes.

## 5 LIMITATIONS

This project provided a scientific base regarding the neuroprotective potentials of progesterone derivatives both using *in-vitro* and *in-vivo* targets. Yet further molecular studies are required to assess the effect of test compounds on the amyloid load, neuro-inflammation and Neurotrophins modulation.

## CONCLUSION

Our study aimed to analyze the neuroprotective effects of progesterone derivatives (AN-1 to AN-5) through *in-vitro* and *in-vivo* studies. Our synthesized compounds exhibited anticholinesterase and DPPH free radical scavenging potential in dose dose-dependent manner. The AN1 was further studied through the behavioral models of amnesia, such as Y-maze, EPM, and open field tests for the assessment of cognitive and anxiolytic potentials of compounds. In behavioral studies, a significant improvement in cognitive performance, learning, memory, and locomotor activities was observed in tested mice. Docking studies demonstrated a high binding affinity of AN1 at the binding site of cholinesterase enzymes. Our results of *in-vitro* as well as *in-vivo* studies support the neuroprotective potentials of progesterone derivatives, and more detailed molecular studies are required for their development as new therapeutics against AD.

## Figures and Tables

**Fig. (2):**
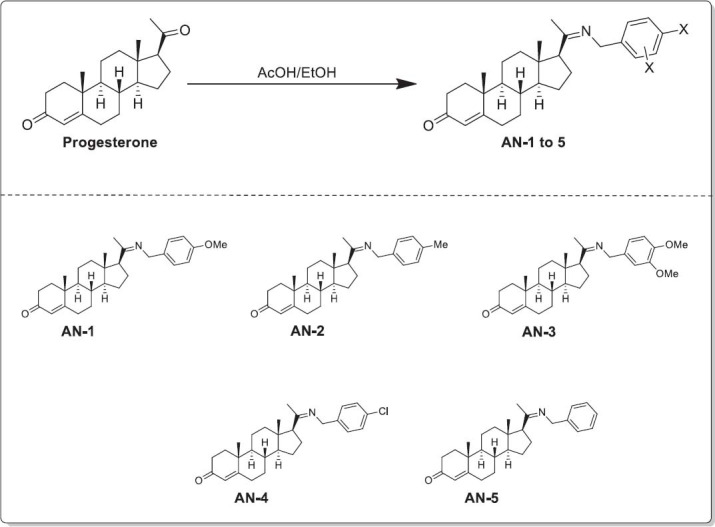
Structures of synthesized derivatives of progesterone (**AN1-AN5**).

**Fig. (3):**
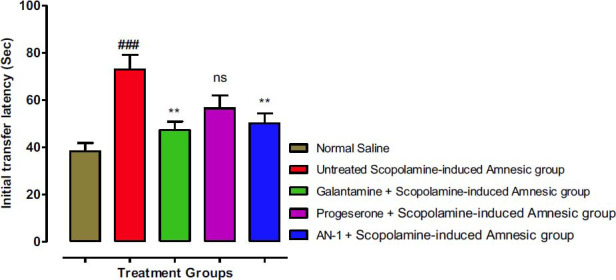
The effects of the AN1 test compound on the ITL using the Elevated Plus Maze (EPM) test. One-way ANOVA followed by Dunnett’s test was applied to the data. The untreated scopolamine-induced amnesia group (Disease group) was compared to the control group (saline-treated normal animals’ group), whereas the other test groups were compared to the disease group. Symbols ** represent *p* value less than 0.01. The value of the scopolamine group was indicated by symbol ### (*p* less than 0.001). ‘ns’ means not significant as compared to the disease group.

**Fig. (4):**
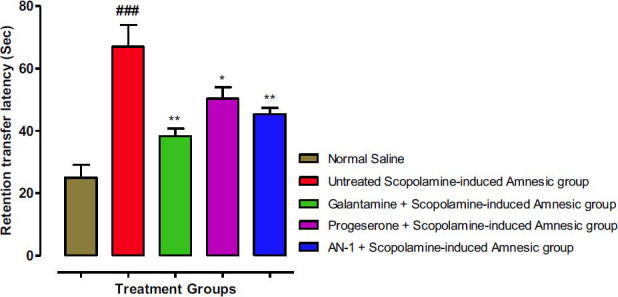
The effects of AN1 test compound on RTL using Elevated Plus Maze (EPM) test. One-way ANOVA followed by Dunnett’s test was applied to the data. The untreated scopolamine-induced amnesia group (Disease group) was compared to the control group (saline-treated normal animals’ group), whereas the other test groups were compared to the disease group. Symbols * and ** represent *p* values less than 0.05 and 0.01 correspondingly. Disease group values were indicated by symbol ### (*p* <0.001).

**Fig. (5):**
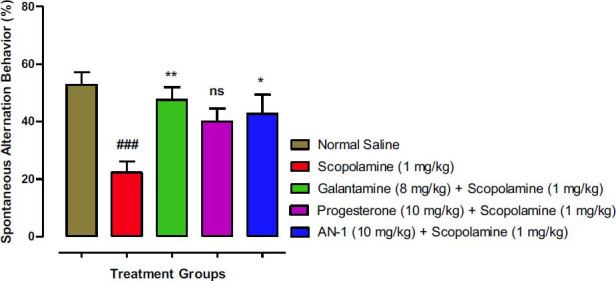
Effects of AN1 compound on spontaneous alternation behavior of mice of various groups in y-maze test. One-way ANOVA followed by Dunnett’s test was applied to the data. Untreated scopolamine-induced amnesia animals (Disease group) were compared to the control group (saline-treated normal animals’ group), whereas the other test groups were compared to the disease group. Symbols * and ** represent *p* values less than 0.05 and 0.01 correspondingly. The value of the scopolamine group was indicated by symbol ### (*p* <0.001). ‘ns’ means not significant as compared to the disease group.

**Fig. (7):**
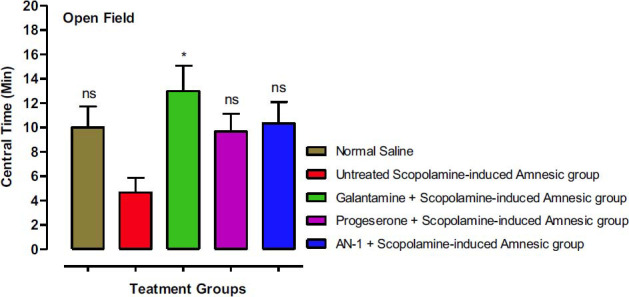
Results regarding the time spent centrally by different groups of mice in OFT. One-way ANOVA followed by Dunnett’s test was applied to the data. The untreated scopolamine-induced amnesia group (Disease group) was compared to the control group (saline-treated normal animals’ group), whereas the other test groups were compared to the disease group. The symbol * represents *p*-value less than 0.05. ‘ns’ means not significant as compared to the disease group.

**Fig. (8):**
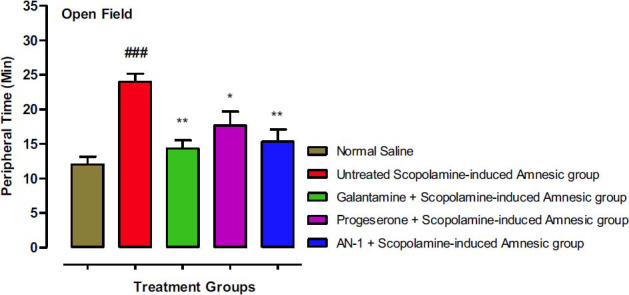
Peripherally spent time by different groups of animals in OFT. One-way ANOVA followed by Dunnett’s test was applied to the data. The untreated scopolamine-induced amnesia group (Disease group) was compared to the control group (saline-treated normal animals’ group), whereas the other test groups were compared to the disease group. Symbols *and ** represent *p* values less than 0.05 and 0.01 correspondingly. The value of the scopolamine group was indicated by symbol ### (*p*<0.001).

**Fig. (10):**
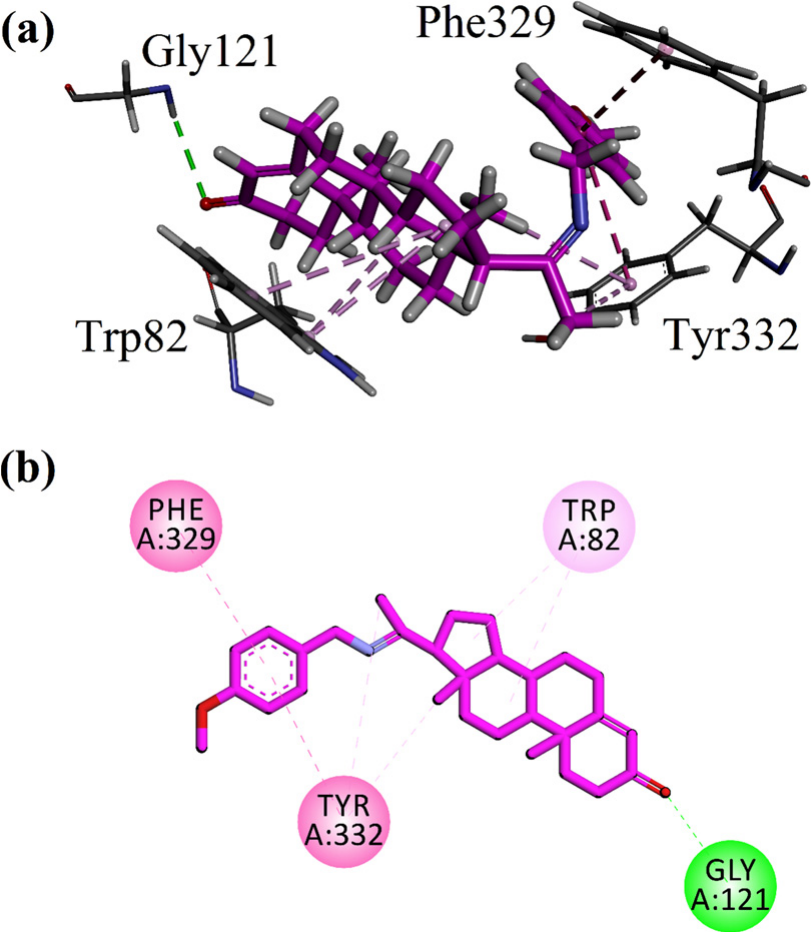
(**a**) 3D (**b**) 2D visualization of the interaction of AN1 compound in the binding site of BChE (PDB ID = 4BDS) after induced fit docking.

**Table 1:** Results of the cholinesterase inhibitory potentials of AN1 to AN5.

Samples	Con. (µg/ml)	% AChE Inhibition	IC_50_ (µM)	**% BChE Inhibition**	IC_50_ (µM)
**AN1**	1000 500 250 125 62.5	88.94 ± 1.20^ns^ 83.22 ± 1.41^ns^ 76.33 ± 4.28^ns^ 69.44 ± 2.43^ns^ 57.94 ± 1,25^ns^	56.52	86.78 ± 1.24^ns^ 80.37 ± 1.38^ns^ 71.94 ± 3.59^ns^ 68.34 ± 2.05^ns^ 61.53 ± 3.24^ns^	43.33
**AN2**	1000 500 250 125 62.5	75.72 ± 2.82*** 75.66 ± 1.26*** 64.27 ± 2.25*** 57.38 ± 3.13*** 48.33 ± 2.83***	173.16	77.46 ± 1.29*** 71.99 ± 4.5*** 74.11 ± 2.83*** 52.90 ± 3.67*** 50.78 ± 2.53***	138.04
**AN3**	1000 500 250 125 62.5	82.82 ± 2.02*** 78.16 ± 2.17*** 64.61 ± 2.94*** 62.94 ± 2.71*** 50.72 ± 1.63***	133.18	87.91 ± 1.96** 81.85 ± 2.58** 64.34 ± 1.40** 59.86 ± 1.50** 55.52 ± 1.65**	111.54
**AN4**	1000 500 250 125 62.5	70.00 ± 2.26*** 67.66 ± 1.74*** 60.27 ± 5.29*** 52.72 ± 2.88*** 47.05 ± 1.58***	213.45	76.33 ± 1.56*** 64.69 ± 1.77*** 63.11 ± 2.00*** 56.85 ± 2.60*** 49.55 ± 1.11***	148.95
**AN5**	1000 500 250 125 62.5	83.50 ± 2.31*** 77.27 ± 1.03*** 65.22 ± 2.81*** 59.72 ± 1.29*** 50.44 ± 2.74***	156.44	85.50 ± 2.83* 75.83 ± 2.05* 71.44 ± 2.54* 65.92 ± 2.50* 58.08 ± 1.58*	63.007
**Galantamine**	1000 500 250 125 62.5	91.88 ± 2.45 86.83 ± 1.26 78.50 ± 2.58 70.27 ± 1.49 64.88 ± 0.64	53.83	89.49 ± 1.93 84.36 ± 1.89 77.36 ± 0.90 72.87 ± 0.42 67.20 ± 0.72	27.31

**Note**: Data values were depicted as mean ± SEM. Two-way ANOVA pursued by the Bonferroni test was applied. In comparison to the standard positive control galantamine, significant differences were as follows: superscript * = “*p”* less than 0.05, ** = “*p”* less than 0.01, and *** = “*p”* less than 0.001, whereas ns = non-significant.

**Table 2:** Results of the DPPH free radicals scavenging potential of synthesized compounds.

Samples	Conc. (µg/ml)	Absorbance	% Inhibition	IC_50_ (µM)
**AN-1**	1000 500 250 125 62.5	0.124 0.178 0.224 0.242 0.325	83.37 ± 2.03** 76.15 ± 2.31** 70.00 ± 3.95** 67.64 ± 3.04** 56.46 ± 1.12**	57.21
**AN-2**	1000 500 250 125 62.5	0.213 0.252 0.256 0.318 0.331	71.47 ± 1.83*** 66.26 ± 1.60*** 65.77 ± 3.32*** 57.44 ± 2.47*** 55.65 ± 2.44***	62.47
**AN-3**	1000 500 250 125 62.5	0.207 0.251 0.290 0.322 0.342	72.32 ± 1.37*** 66.44 ± 2.58*** 61.18 ± 2.23*** 56.86 ± 2.33*** 54.18 ± 2.16***	86.37
**AN-4**	1000 500 250 125 62.5	0.225 0.248 0.294 0.332 0.397	69.87 ± 2.81*** 66.80 ± 3.47*** 60.69 ± 1.92*** 55.52 ± 3.43*** 46.88 ± 0.65***	188.20
**AN-5**	1000 500 250 125 62.5	0.201 0.243 9.258 0.321 0.357	73.12 ± 1.04*** 67.51 ± 2.56*** 65.46 ± 1.83*** 56.99 ± 2.50*** 52.18 ± 1.42***	110.45
**Ascorbic Acid**	1000 500 250 125 62.5	0.066 0.104 0.190 0.215 0.241	91.17 ± 1.62 86.00 ± 0.70 74.50 ± 3.04 71.25 ± 4.29 67.69 ± 2.18	54.05

**Note**: Data values were depicted as mean ± SEM. Two-way ANOVA followed by the Bonferroni test was applied. In comparison to the standard positive control ascorbic acid, significant differences were as follows: superscript ** = “*p”* less than 0.01 and *** = “*p”* less than 0.001.

## Data Availability

The data and supportive information are available within the article.

## LIST OF ABBREVIATIONS

AChE: Acetylcholinesterase
AD: Alzheimer's Disease
APP: Amyloid Precursor Protein
BChE: Butyrylcholinesterase
CNS: Central Nervous System
EPM: Elevated Plus Maze
FAD: Familial Alzheimer’s Disease
MAO: Monoamine Oxidase
NFTs: Neurofibrillary Tangles
ROS: Reactive Oxygen Species
SPs: Senile Plaques

## SUPPLEMENTARY MATERIAL

Supplementary material containing compounds scheme (Fig. **S1**), ^1^H NMR spectrum of **AN1** compound (Fig. **S2**), ^13^C NMR spectrum of **AN1** compound (Fig. **S3**), ^1^H NMR spectrum of **AN2** compound (Fig. **S4**), ^13^C NMR spectrum of **AN2** compound (Fig. **S5**), ^1^H NMR spectrum of **AN5** compound (Fig. **S6**) and ^13^C NMR spectrum of **AN5** compound (Fig. **S7**) is available on the publisher’s website along with the published article.

## References

[1] Ayaz M., Junaid M., Ullah F., Subhan F., Sadiq A., Ali G., Ovais M., Shahid M., Ahmad A., Wadood A., El-Shazly M., Ahmad N., Ahmad S. (2017). Anti-Alzheimer’s studies on β-sitosterol isolated from Polygonum hydropiper L.. Front. Pharmacol..

[2] Dubois B., Feldman H.H., Jacova C., Cummings J.L., DeKosky S.T., Barberger-Gateau P., Delacourte A., Frisoni G., Fox N.C., Galasko D., Gauthier S., Hampel H., Jicha G.A., Meguro K., O’Brien J., Pasquier F., Robert P., Rossor M., Salloway S., Sarazin M., de Souza L.C., Stern Y., Visser P.J., Scheltens P. (2010). Revising the definition of Alzheimer’s disease: A new lexicon.. Lancet Neurol..

[3] Wimo A., Guerchet M., Ali G.C., Wu Y.T., Prina A.M., Winblad B., Jönsson L., Liu Z., Prince M. (2017). The worldwide costs of dementia 2015 and comparisons with 2010.. Alzheimers Dement..

[4] Frisoni G.B., Boccardi M., Barkhof F., Blennow K., Cappa S., Chiotis K., Démonet J.F., Garibotto V., Giannakopoulos P., Gietl A., Hansson O., Herholz K., Jack C.R., Nobili F., Nordberg A., Snyder H.M., Ten Kate M., Varrone A., Albanese E., Becker S., Bossuyt P., Carrillo M.C., Cerami C., Dubois B., Gallo V., Giacobini E., Gold G., Hurst S., Lönneborg A., Lovblad K.O., Mattsson N., Molinuevo J.L., Monsch A.U., Mosimann U., Padovani A., Picco A., Porteri C., Ratib O., Saint-Aubert L., Scerri C., Scheltens P., Schott J.M., Sonni I., Teipel S., Vineis P., Visser P.J., Yasui Y., Winblad B. (2017). Strategic roadmap for an early diagnosis of Alzheimer’s disease based on biomarkers.. Lancet Neurol..

[5] Mushtaq A., Anwar R., Ahmad M. (2018). Lavandula stoechas L alleviates dementia by preventing oxidative damage of cholinergic neurons in mice brain.. Trop. J. Pharm. Res..

[6] Rossor M.N., Fox N.C., Freeborough P.A., Harvey R.J. (1996). Clinical features of sporadic and familial Alzheimer’s disease.. Neurodegeneration.

[7] Selkoe D.J. (2001). Alzheimer’s disease: Genes, proteins, and therapy.. Physiol. Rev..

[8] Reisberg B., Doody R., Stöffler A., Schmitt F., Ferris S., Möbius H.J. (2003). Memantine in moderate-to-severe Alzheimer’s disease.. N. Engl. J. Med..

[9] Minati L., Edginton T., Grazia B.M., Giaccone G. (2009). Current concepts in Alzheimer’s disease: A multidisciplinary review.. Am. J. Alzheimers Dis. Other Demen..

[10] Selkoe D.J., Hardy J. (2016). The amyloid hypothesis of Alzheimer’s disease at 25 years.. EMBO Mol. Med..

[11] De Ferrari G.V., Canales M.A., Shin I., Weiner L.M., Silman I., Inestrosa N.C. (2001). A structural motif of acetylcholinesterase that promotes amyloid beta-peptide fibril formation.. Biochemistry.

[12] Yan M.H., Wang X., Zhu X. (2013). Mitochondrial defects and oxidative stress in Alzheimer disease and Parkinson disease.. Free Radic. Biol. Med..

[13] Behl C., Moosmann B. (2002). Antioxidant neuroprotection in Alzheimer’s disease as preventive and therapeutic approach.. Free Radic. Biol. Med..

[14] Birks J.S. (2006). Cholinesterase inhibitors for Alzheimer’s disease.. Cochrane Database Syst. Rev..

[15] Anand P., Singh B. (2013). A review on cholinesterase inhibitors for Alzheimer’s disease.. Arch. Pharm. Res..

[16] Bond M., Rogers G., Peters J., Anderson R., Hoyle M., Miners A., Moxham T., Davis S., Thokala P., Wailoo A., Jeffreys M., Hyde C. (2012). The effectiveness and cost-effectiveness of donepezil, galantamine, rivastigmine and memantine for the treatment of Alzheimer’s disease (review of Technology Appraisal No. 111): A systematic review and economic model.. Health Technol. Assess..

[17] Huang Y., Mucke L. (2012). Alzheimer mechanisms and therapeutic strategies.. Cell.

[18] Melcangi R.C., Garcia-Segura L.M., Mensah-Nyagan A.G. (2008). Neuroactive steroids: State of the art and new perspectives.. Cell. Mol. Life Sci..

[19] Borah P., Banik B.K. (2020). Diverse synthesis of medicinally active steroids.. Green Approaches in Medicinal Chemistry for Sustainable Drug Design..

[20] Mayo W., Lemaire V., Malaterre J., Rodriguez J.J., Cayre M., Stewart M.G., Kharouby M., Rougon G., Le Moal M., Piazza P.V., Abrous D.N. (2005). Pregnenolone sulfate enhances neurogenesis and PSA-NCAM in young and aged hippocampus.. Neurobiol. Aging.

[21] Singh M., Sumien N., Kyser C., Simpkins J.W. (2008). Estrogens and progesterone as neuroprotectants: What animal models teach us.. Front. Biosci..

[22] Herson P.S., Koerner I.P., Hurn P.D. (2009). Sex, sex steroids, and brain injury.. Seminars in reproductive medicine..

[23] He H., Kulanthaivel P., Baker B.J., Kalter K., Darges J., Cofield D., Wolff L., Adams L. (1995). New antiproliferative and antiinflammatory 9,11-secosterols from the gorgonian Pseudopterogorgia sp.. Tetrahedron.

[24] Roglio I., Bianchi R., Gotti S., Scurati S., Giatti S., Pesaresi M., Caruso D., Panzica G.C., Melcangi R.C. (2008). Neuroprotective effects of dihydroprogesterone and progesterone in an experimental model of nerve crush injury.. Neuroscience.

[25] Leonelli E., Bianchi R., Cavaletti G., Caruso D., Crippa D., Garcia-Segura L.M., Lauria G., Magnaghi V., Roglio I., Melcangi R.C. (2007). Progesterone and its derivatives are neuroprotective agents in experimental diabetic neuropathy: A multimodal analysis.. Neuroscience.

[26] Perry E.K. (1986). The cholinergic hypothesis--ten years on.. Br. Med. Bull..

[27] Schwarz M., Glick D., Loewenstein Y., Soreq H. (1995). Engineering of human cholinesterases explains and predicts diverse consequences of administration of various drugs and poisons.. Pharmacol. Ther..

[28] Amin M.J., Miana G.A., Rashid U., Rahman K.M., Khan H., Sadiq A. (2020). SAR based in-vitro anticholinesterase and molecular docking studies of nitrogenous progesterone derivatives.. Steroids.

[29] Jabeen M., Choudhry M.I., Miana G.A., Rahman K.M., Rashid U.; (2018). Khan, H.; Arshia, ; Sadiq, A. Synthesis, pharmacological evaluation and docking studies of progesterone and testosterone derivatives as anticancer agents.. Steroids.

[30] Trevisan M.T.S., Macedo Fb. (2003). V.V.; Meent M.; Rhee, I.K.; Verpoorte, R. Screening for acetylcholinesterase inhibitors from plants to treat Alzheimer’s disease.. QuÃmica Nova.

[31] Ellman G.L., Courtney K.D., Andres V., Featherstone R.M. (1961). A new and rapid colorimetric determination of acetylcholinesterase activity.. Biochem. Pharmacol..

[32] Kamal Z., Ullah F., Ayaz M., Sadiq A., Ahmad S., Zeb A., Hussain A., Imran M. (2015). Anticholinesterse and antioxidant investigations of crude extracts, subsequent fractions, saponins and flavonoids of Atriplex laciniata L. potential effectiveness in Alzheimer’s and other neurological disorders.. Biol. Res..

[33] Nicklas W., Baneux P., Boot R., Decelle T., Deeny A.A., Fumanelli M., Illgen-Wilcke B. (2002). Recommendations for the health monitoring of rodent and rabbit colonies in breeding and experimental units.. Lab. Anim..

[34] Kilkenny C., Browne W., Cuthill I.C., Emerson M., Altman D.G. (2010). Animal research: Reporting in vivo experiments: The ARRIVE guidelines.. Br. J. Pharmacol..

[35] Ahmad N., Subhan F., Islam N.U., Shahid M., Rahman F.U., Sewell R.D.E. (2017). Gabapentin and its salicylaldehyde derivative alleviate allodynia and hypoalgesia in a cisplatin-induced neuropathic pain model.. Eur. J. Pharmacol..

[36] Hughes R.N. (2004). The value of spontaneous alternation behavior (SAB) as a test of retention in pharmacological investigations of memory.. Neurosci. Biobehav. Rev..

[37] Jeon S.J.K. (2017). The ameliorating effect of 1-palmitoyl-2-linoleoyl-3-acetylglycerol on scopolamine-induced memory impairment via acetylcholinesterase inhibition and LTP activation.. Behav. Brain Res..

[38] Bhuvanendran S., Kumari Y., Othman I., Shaikh M.F. (2018). Amelioration of cognitive deficit by embelin in a scopolamine-induced Alzheimer’s disease-like condition in a rat model.. Front. Pharmacol..

[39] Lu C., Dong L., Lv J., Wang Y., Fan B., Wang F., Liu X. (2018). 20(S)-protopanaxadiol (PPD) alleviates scopolamine-induced memory impairment via regulation of cholinergic and antioxidant systems, and expression of Egr-1, c-Fos and c-Jun in mice.. Chem. Biol. Interact..

[40] Choleris E., Thomas A.W., Kavaliers M., Prato F.S. (2001). A detailed ethological analysis of the mouse open field test: Effects of diazepam, chlordiazepoxide and an extremely low frequency pulsed magnetic field.. Neurosci. Biobehav. Rev..

[41] Shahidul I.M., Mohammed Al-Majid A., Nageh S.E., Yousuf S., Ayaz M., Nawaz A., Wadood A., Rehman A.U., Prakash V.V., Motiur R.A.F.M., Barakat A. (2022). Synthesis of spiro‐oxindole analogs engrafted pyrazole scaffold as potential Alzheimer’s disease therapeutics: Anti‐oxidant, enzyme inhibitory and molecular docking approaches.. ChemistrySelect.

[42] Ayaz M., Mosa O.F., Nawaz A., Hamdoon A.A.E., Elkhalifa M.E.M., Sadiq A., Ullah F., Ahmed A., Kabra A., Khan H., Murthy H.C.A. (2024). Neuroprotective potentials of lead phytochemicals against Alzheimer’s disease with focus on oxidative stress-mediated signaling pathways: Pharmacokinetic challenges, target specificity, clinical trials and future perspectives.. Phytomedicine.

[43] Alhasaniah A.H., Ahmad Z., Zeb A., Mahnashi M.H., Sadiq A., Ayaz M. (2023). Polarity directed solvent extracts from Bukiniczia Cabulica (Boiss.) Lincz. ameliorate scopolamine induced amnesia: HPLC-DAD polyphenolics analysis, cholinesterase, COX2, BACE1 inhibitory, anti-amyloid, antioxidant, molecular docking and behavioral correlates.. J. Mol. Liq..

[44] Alam A., Ali G., Nawaz A., AlOmar T.S., Rauf A., Ayaz M., Ahmad S., Almasoud N., AlOmar A.S., Khalil A.A., Wilairatana P. (2023). Neuroprotective evaluation of diospyrin against drug-induced Alzheimer’s disease.. Fitoterapia.

[45] Mahnashi M.H., Ashraf M., Alhasaniah A.H., Ullah H., Zeb A., Ghufran M., Fahad S., Ayaz M., Daglia M. (2023). Polyphenol-enriched Desmodium elegans DC. ameliorate scopolamine-induced amnesia in animal model of Alzheimer’s disease: In vitro, in vivo and in silico approaches.. Biomed. Pharmacother..

[46] Ferreira-Vieira T.H., Guimaraes I.M., Silva F.R., Ribeiro F.M. (2016). Alzheimer’s disease: Targeting the cholinergic system.. Curr. Neuropharmacol..

[47] Nazir N., Karim N., Abdel-Halim H., Khan I., Wadood S.F., Nisar M. (2018). Phytochemical analysis, molecular docking and antiamnesic effects of methanolic extract of Silybum marianum (L.) Gaertn seeds in scopolamine induced memory impairment in mice.. J. Ethnopharmacol..

[48] Blokland A. (1995). Acetylcholine: A neurotransmitter for learning and memory?. Brain Res. Brain Res. Rev..

[49] Budzynska B., Boguszewska-Czubara A., Kruk-Slomka M., Skalicka-Wozniak K., Michalak A., Musik I., Biala G. (2015). Effects of imperatorin on scopolamine-induced cognitive impairment and oxidative stress in mice.. Psychopharmacology.

[50] Taylor P. (2011). Anticholinesterase agents. Goodman & Gilman’s The Pharmacological Basis of Therapeutics..

[51] Darreh-Shori T., Hellström-Lindahl E., Flores-Flores C., Guan Z.Z., Soreq H., Nordberg A. (2004). Long‐lasting acetylcholinesterase splice variations in anticholinesterase‐treated Alzheimer’s disease patients.. J. Neurochem..

[52] Voet D., Voet J.G. (1995). Serine proteases.. Biochemistry..

[53] (2004). Atta-Ur-Rahman.; Atia-Tul-Wahab.; Nawaz, S.A.; Choudhary, M.I. New cholinesterase inhibiting bisbenzylisoquinoline alkaloids from Cocculus pendulus.. Chem. Pharm. Bull..

[54] Ahmad W., Ahmad B., Ahmad M., Iqbal Z., Nisar M., Ahmad M. (2003). In vitro inhibition of acetylcholinesterase, buty-rylcholinesterase and lipoxygenase by crude extract of Myricaria elegans Royle.. J. Biol. Sci..

[55] Coyle J., Kershaw P. (2001). Galantamine, a cholinesterase inhibitor that allosterically modulates nicotinic receptors: Effects on the course of Alzheimer’s disease.. Biol. Psychiatry.

[56] Zahoor M., Shafiq S., Ullah H., Sadiq A., Ullah F. (2018). Isolation of quercetin and mandelic acid from Aesculus indica fruit and their biological activities.. BMC Biochem..

[57] Tong X., Li X., Ayaz M., Ullah F., Sadiq A., Ovais M., Shahid M., Khayrullin M., Hazrat A. (2021). Neuroprotective studies on Polygonum hydropiper L. essential oils using transgenic animal models.. Front. Pharmacol..

[58] Zeb A., Hassan M., Ayaz M. (2024). Carotenoid and phenolic profiles and antioxidant and anticholinesterase activities of leaves and berries of Phytolacca acinosa.. ACS Food Sci. Technol..

[59] Conrad C.D., Lupien S.J., Thanasoulis L.C., McEwen B.S. (1997). The effects of Type I and Type II corticosteroid receptor agonists on exploratory behavior and spatial memory in the Y-maze.. Brain Res..

[60] Martin S., Jones M., Simpson E., van den Buuse M. (2003). Impaired spatial reference memory in aromatase-deficient (ArKO) mice.. Neuroreport.

[61] Waseem W., Anwar F., Saleem U., Ahmad B., Zafar R., Anwar A., Saeed Jan M., Rashid U., Sadiq A., Ismail T. (2022). Prospective evaluation of an amide-based zinc scaffold as an anti-alzheimer agent: in vitro, in vivo, and computational studies.. ACS Omega.

[62] Ferretti L., McCurry S.M., Logsdon R., Gibbons L., Teri L. (2001). Anxiety and Alzheimer’s disease.. J. Geriatr. Psychiatry Neurol..

[63] Mahnashi M.H., Ayaz M., Ghufran M., Almazni I.A., Alqahtani O., Alyami B.A., Alqahtani Y.S., Khan H.A., Sadiq A., Waqas M. (2023). Phytochemicals-based β-amyloid cleaving enzyme-1 and MAO-B inhibitors for the treatment of Alzheimer’s disease: Molecular simulations-based predictions.. J. Biomol. Struct. Dyn..

[64] Inestrosa N.C., Alvarez A., Pérez C.A., Moreno R.D., Vicente M., Linker C., Casanueva O.I., Soto C., Garrido J. (1996). Acetylcholinesterase accelerates assembly of amyloid-β-peptides into Alzheimer’s fibrils: Possible role of the peripheral site of the enzyme.. Neuron.

[65] Mahnashi M.H., Alshahrani M.A., Nahari M.H., Hassan S.S., Jan M.S., Ayaz M., Ullah F., Alshehri O.M., Alshehri M.A., Rashid U., Sadiq A. (2022). In-vitro, in-vivo, molecular docking and ADMET studies of 2-substituted 3,7-dihydroxy-4H-chromen-4-one for oxidative stress, inflammation and Alzheimer’s disease.. Metabolites.

[66] Ayaz M., Wadood A., Sadiq A., Ullah F., Anichkina O., Ghufran M. (2022). In-silico evaluations of the isolated phytosterols from polygonum hydropiper L against BACE1 and MAO drug targets.. J. Biomol. Struct. Dyn..

