# Supplementary material for: Synthetic Derivates of Progesterone Ameliorate Scopolamine-Induced Cognitive Deficits in Animal Models: Antioxidant, Enzyme Inhibitory, Molecular Docking and Behavioral Correlates
Source: Curr Neuropharmacol. 2025 Mar 27;23(13):1797–812. doi: 10.2174/011570159X357722250212094900 (PMC12645137; doi:10.2174/011570159X357722250212094900)
Supplement: Supplementary file 1 — Supplementary material containing compounds scheme (Fig. S1), 1H NMR spectrum of AN1 compound (Fig. S2), 13C NMR spectrum of AN1 compound (Fig. S3), 1H NMR spectrum of AN2 compound (Fig. S4), 13C NMR spectrum of AN2 compound (Fig. S5), 1H NMR spectrum of AN5 compound (Fig. S6) and 13C NMR spectrum of AN5 compound (Fig. S7) is available on the publisher’s website along with the published article. [file CN-23-13-1797_SD1.pdf]

Supplementary Material

Synthetic Derivates of Progesterone Ameliorate Scopolamine-Induced Cognitive Deficits in Animal Models: Antioxidant, Enzyme Inhibitory, Molecular Docking and Behavioral Correlates

Asif Nawaz[1], Abdul Sadiq[1,*], Nasreena Bashir[2], Umer Rashid[3], Farhat Ullah[1], Shahbaz Khan[4], Farman Ullah[5], Mohammad Inam Khan[6] and Muhammad Ayaz[1,*]

[1]Department of Pharmacy, Faculty of Biological Sciences, University of Malakand, Chakdara, 18800 Dir (L), Khyber Pakhtunkhwa, Pakistan; [2]Department of Clinical Laboratory Sciences, College of Applied Medical Sciences, King Khalid University, Abha, 61421, Saudi Arabia; [3]Department of Chemistry, COMSATS University Islamabad, 22060 Abbottabad, Pakistan; [4]Khyber Medical College, Khyber Medical University, Peshawar, Pakistan; [5]Department of Pharmacy, Kohat University of Science and Technology (KUST), Khyber Pakhtunkhwa (KP), Pakistan; [6]Public Health Department, College of Health Sciences, Saudi Electronic University, Abha Male 61421, Saudi Arabia

1875-6190/25 $65.00+.00 **© 2025 Bentham Science Publishers**

Synthesis of Progesterone Derivatives (AN1-AN5)

Synthesis of progesterone derivatives were performed by reacting progesterone with commercially available amine in the presence of catalytic amount of acetic acid in ethanol to catalyse the reaction. The reactions were monitored using thin layer chromatography. At completion, the reaction products precipitated out. The synthesized compounds structures were confirmed by ^{1}H and ^{13}C NMR analysis.

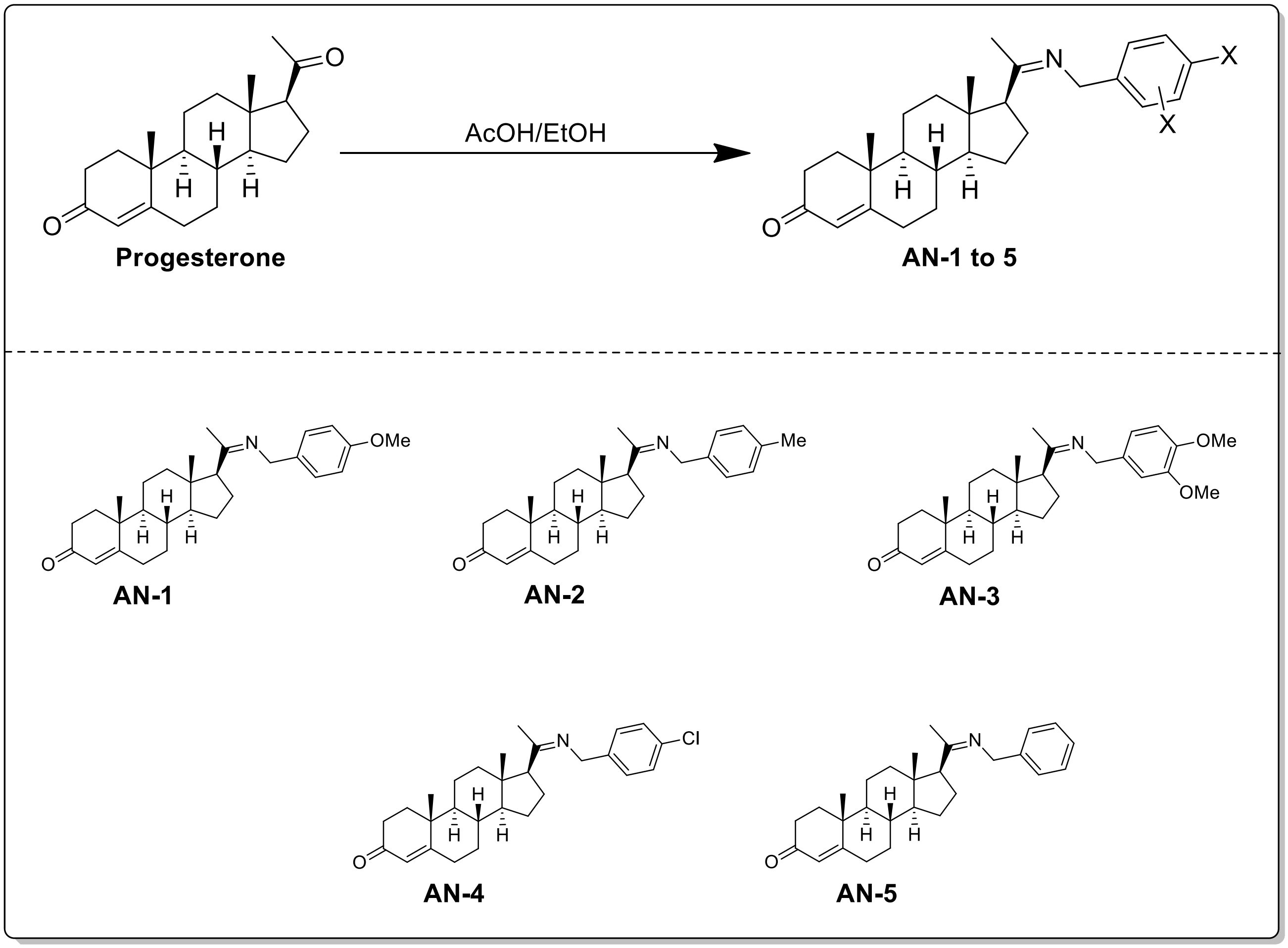

Figure S1: Synthesis scheme of the Progesterone derivatives.

Synthesis of compound AN1

Synthesis of compound AN-1 was performed by reacting progesterone (1 equivalent, 1mmol, 314.46 mg) with 4-Methoxybenzylamine (1 equivalent, 1mmol, 137.18 mg) in the existence of acetic acid (catalytic amount) (20 mol%, 11.45 µl) in ethanol (1 M, 1 ml). The reaction was completed in 4 hours.

Synthesis of compound AN-2

Synthesis of compound AN-2 was performed by reacting progesterone (1 equivalent, 1mmol, 314.46 mg) with 4-Methylbenzylamine (1 equivalent, 1mmol, 121.18 mg) in the existence acetic acid (catalytic amount) (20 mol%, 11.45 µl) in ethanol (1 M, 1 ml). The reaction was completed in 5 hours.

Synthesis of compound AN-3

Synthesis of compound AN-3 was performed by reacting progesterone (1 equivalent, 1mmol, 314.46 mg) with 3,4-Dimethoxybenzylamine (1 equivalent, 1mmol, 167.21 mg) in the existence of acetic acid (catalytic amount) (20 mol%, 11.45 µl) in ethanol (1 M, 1 ml). The reaction was completed in 8 hours.

Synthesis of compound AN-4

Synthesis of compound AN-4 was performed by reacting progesterone (1 equivalent, 1mmol, 314.46 mg) with 4-Chlorobenzylamine (1 equivalent, 1mmol, 141.60 mg) in the existence of acetic acid (catalytic amount) (20 mol%, 11.45 µl) in ethanol (1 M, 1 ml). The reaction was completed in 8 hours.

Synthesis of compound AN-5

Synthesis of compound AN-5 was performed by reacting progesterone (1 equivalent, 1mmol, 314.46 mg) with Benzylamine (1 equivalent, 1mmol, 107.15 mg) in the existence of acetic acid (catalytic amount) (20 mol%, 11.45 µl) in ethanol (1 M, 1 ml). The reaction was completed in 4 hours.

Compound AN1

The chemical name of compound **AN1** is (8S,9S,10R,13S,14S)-17-((Z)-1-((4-methoxybenzyl)imino)ethyl)-10,13-dimethyl-1,2,6,7,8,9,10,11,12,13,14,15,16,17-tetradecahydro-3H-cyclopenta[a]phenanthren-3-one. The isolated yield of the compound was 87% with Rf value of 0.32 (solvent system was 70:30 n-hexane and ethyl acetate). The ^{1}H NMR of compound **AN1** is shown in **Figure S2**. ^{1}H NMR (chloroform D, 400 MHz): 7.12 (d, J = 8.61 Hz, 2H), 6.87 (d, J = 8.58 Hz, 2H), 5.76 (s, 1H), 4.56 (s, 2H), 3.84 (s, 3H), 2.53 (t, J = 9.05 Hz, 1H), 2.47-2.17 (m, 5H), 2.16-2.13 (m, 1H), 2.12 (s, 3H), 2.07-2.01 (m, 2H), 1.89-1.83 (m, 1H), 1.75-1.61 (m, 5H), 1.54-1.40 (m, 2H), 1.32-1.22 (m, 1H), 1.18 (m, 3H), 1.16-0.95 (m, 2H), 0.67 (s, 3H). The ^{13}C NMR of compound **AN1** is shown in **Figure S** . ^{13}C NMR (chloroform D, 100 MHz): 201.00, 173.08, 171.25, 155.35, 132.50, 128.75, 127.97, 126.59, 56.95, 51.60, 43.59, 42.45, 36.78, 35.39, 33.35, 31.30, 28.83, 28.28, 27.93, 26.42, 25.22, 23.78, 21.78, 21.48, 20.22, 18.18, 16.61 and 14.87.

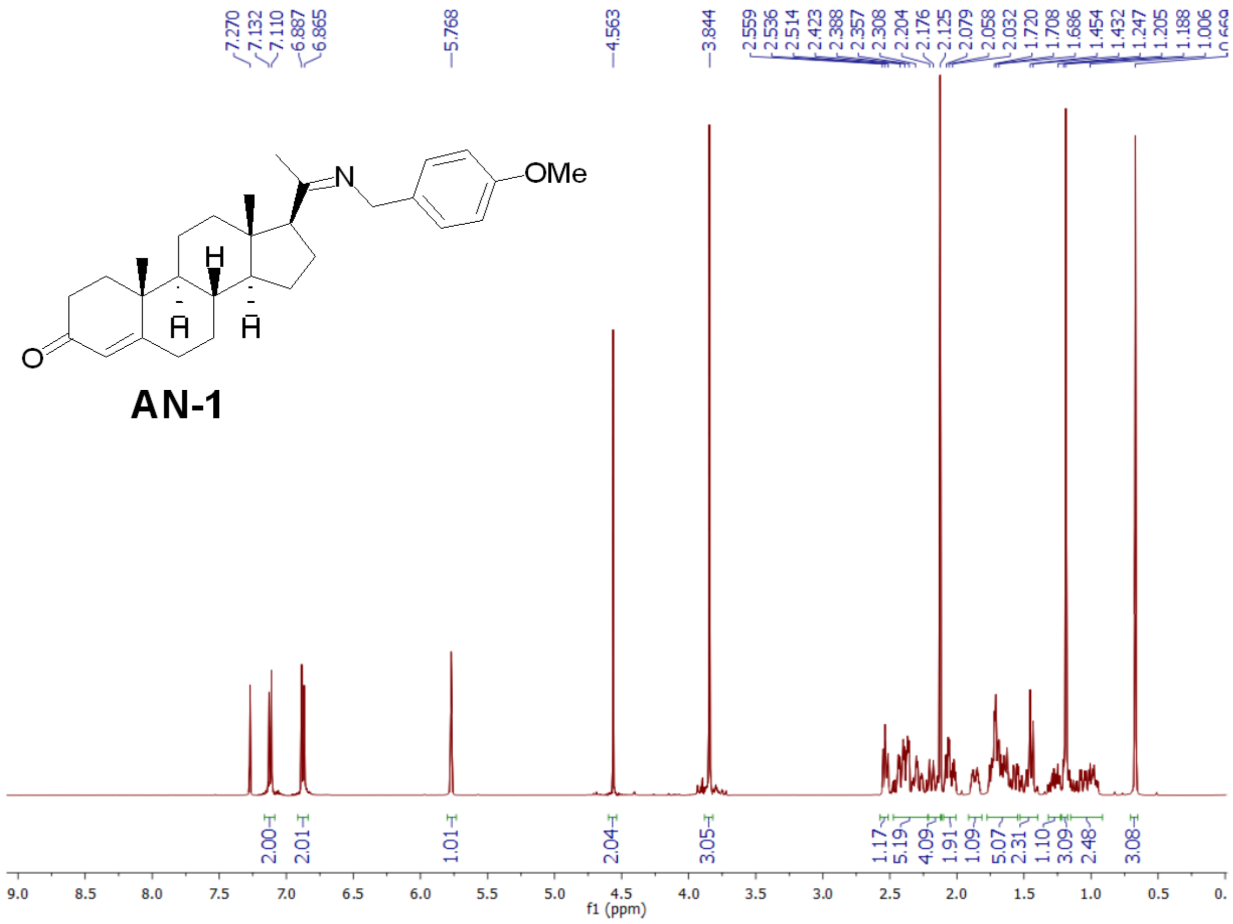

Figure S2: [1]**H NMR spectrum of AN1 compound.**

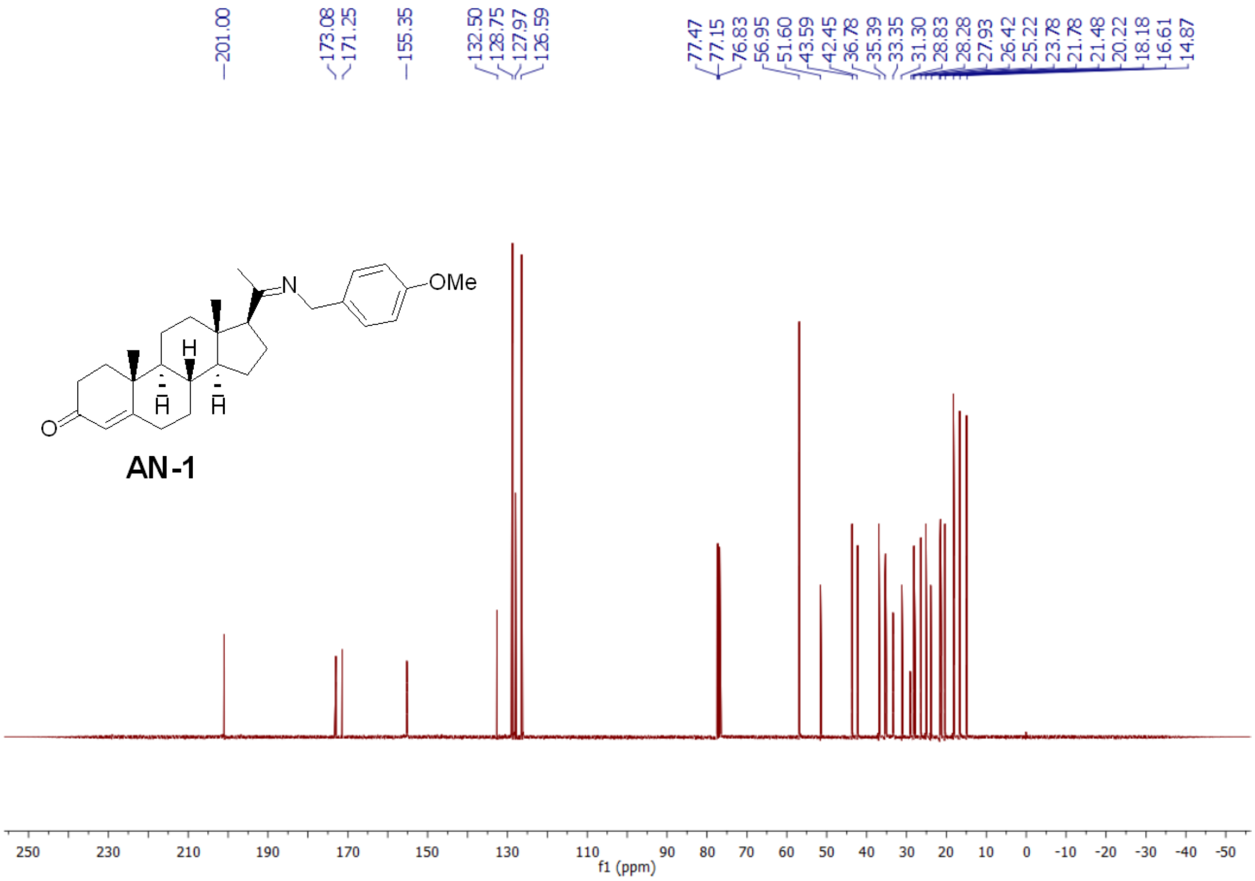

Figure S3: ^{13}C NMR spectrum of AN1 compound.

Compound AN2

The chemical name of compound **AN2** is (8S,9S,10R,13S,14S)-17-((Z)-1-((4-methylbenzyl)imino)ethyl)-10,13-dimethyl-1,2,6,7,8,9,10,11,12,13,14,15,16,17-tetradecahydro-3H-cyclopenta[a]phenanthren-3-one. The isolated yield of the compound was 84% with Rf value of 0.38 (solvent system was 70:30 n-hexane and ethyl acetate). The ^{1}H NMR of compound **AN2** is shown in **Figure S4**. ^{1}H NMR (chloroform D, 400 MHz): 7.10 (d, J = 8.51 Hz, 2H), 6.98 (d, J = 8.50 Hz, 2H), 5.73 (s, 1H), 4.54 (s, 2H), 2.54 (t, J = 8.74 Hz, 1H), 2.46-2.30 (m, 5H), 2.23 (s, 3H), 2.12 (s, 3H), 2.07-2.02 (m, 2H), 1.89-1.84 (m, 1H), 1.75-1.23 (m, 9H), 1.18 (m, 3H), 1.13-0.97 (m, 2H), 0.81 (s, 3H). The ^{13}C NMR of compound **AN2** is shown in **Figure S5**. ^{13}C NMR (chloroform D, 100 MHz): 202.20, 170.04, 168.12, 156.26, 131.93, 129.20, 128.07, 126.70, 52.81, 44.20, 42.43, 35.81, 34.86, 32.67, 32.03, 31.31, 29.38, 28.81, 26.31, 25.26, 23.67, 22.93, 22.45, 21.47, 17.38, 16.09 and 13.67.

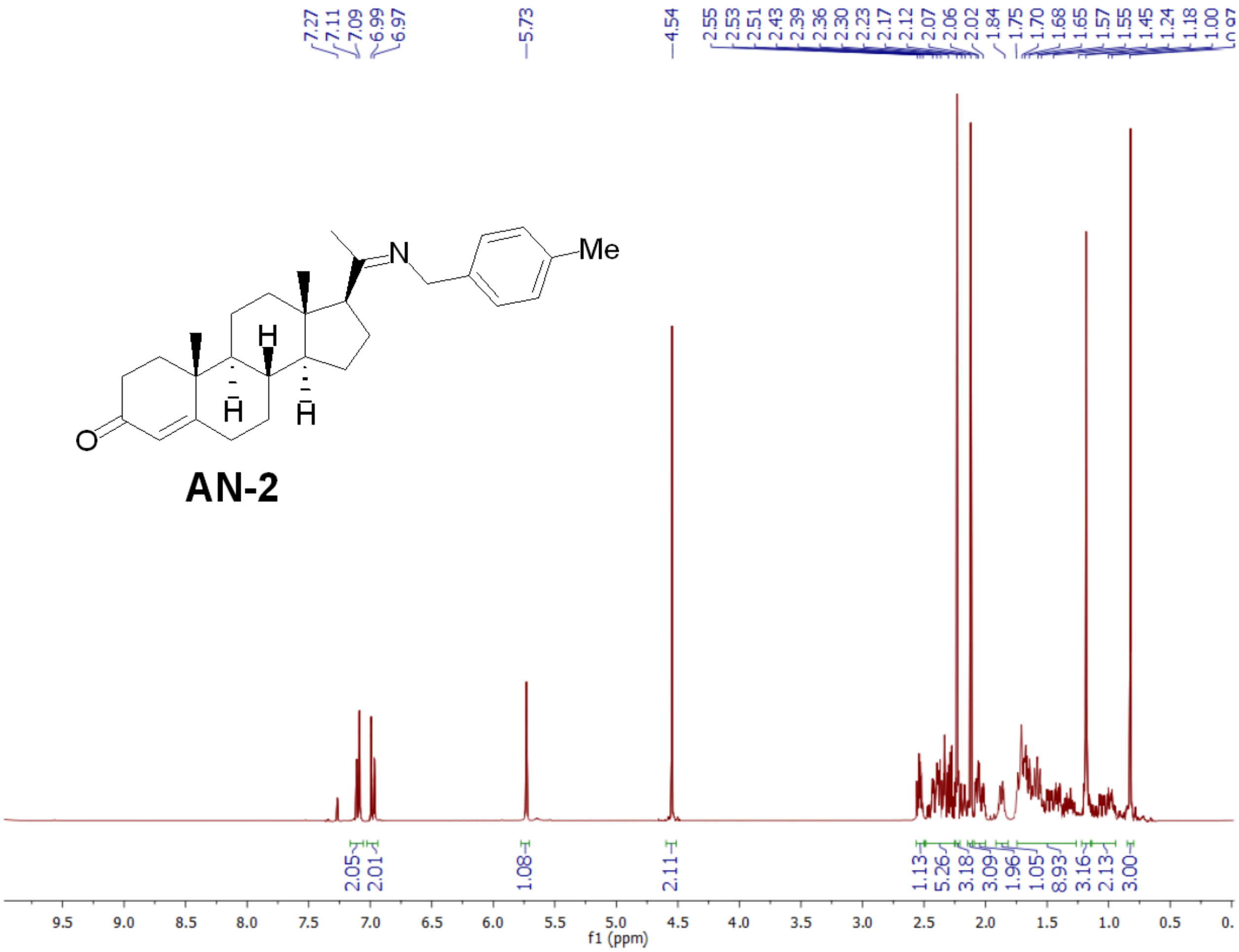

Figure S4: ^{1}H NMR spectrum of AN2 compound.

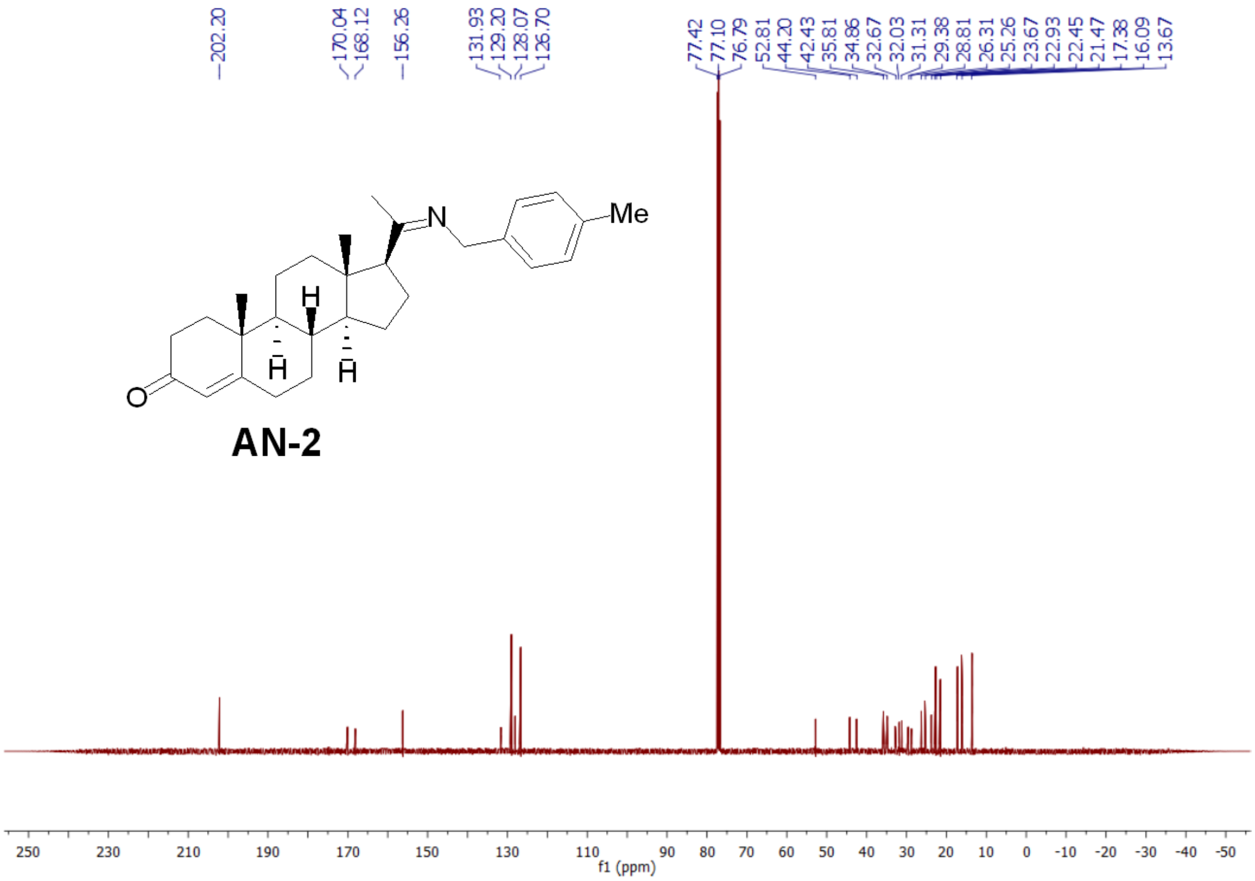

Figure S5: ^{13}C NMR spectrum of AN2 compound.

Compound AN5

The chemical name of compound **AN5** is (8S,9S,10R,13S,14S)-17-((Z)-1-(benzylimino)ethyl)-10,13-dimethyl-1,2,6,7,8,9,10,11,12,13,14,15,16,17-tetradecahydro-3H cyclopenta[a]phenanthren-3-one. The isolated yield of the compound was 92% with Rf value of 0.42 (solvent system was 70:30 n-hexane and ethyl acetate). The ^{1}H NMR of compound **AN5** is shown in **Figure S6**. ^{1}H NMR (chloroform D, 400 MHz): 7.54-7.46 (m, 3H), 7.40-7.33 (m, 2H), 5.38 (bs, 1H), 2.84 (t, J = 8.91 Hz, 1H), 2.41-2.21 (m, 3H), 2.06-1.94 (m, 2H), 1.92 (s, 3H), 1.88-1.81 (m, 2H), 1.78-1.68 (m, 2H), 1.64-1.42 (m, 9H), 1.38-1.23 (m, 2H), 1.17-1.03 (m, 1H), 1.01 (s, 3H), 0.72 (s, 3H). The ^{13}C NMR of compound **AN5** is shown in **Figure S7**. ^{13}C NMR (chloroform D, 100 MHz): 202.85, 171.97, 170.97, 135.87, 128.79, 128.72, 128.18, 128.08, 56.78, 52.19, 50.44, 43.18, 42.60, 40.20, 36.73, 35.23, 34.43, 33.50, 31.50, 30.29, 29.48, 27.73, 25.80, 21.20, 18.43, 16.75 and 14.36.

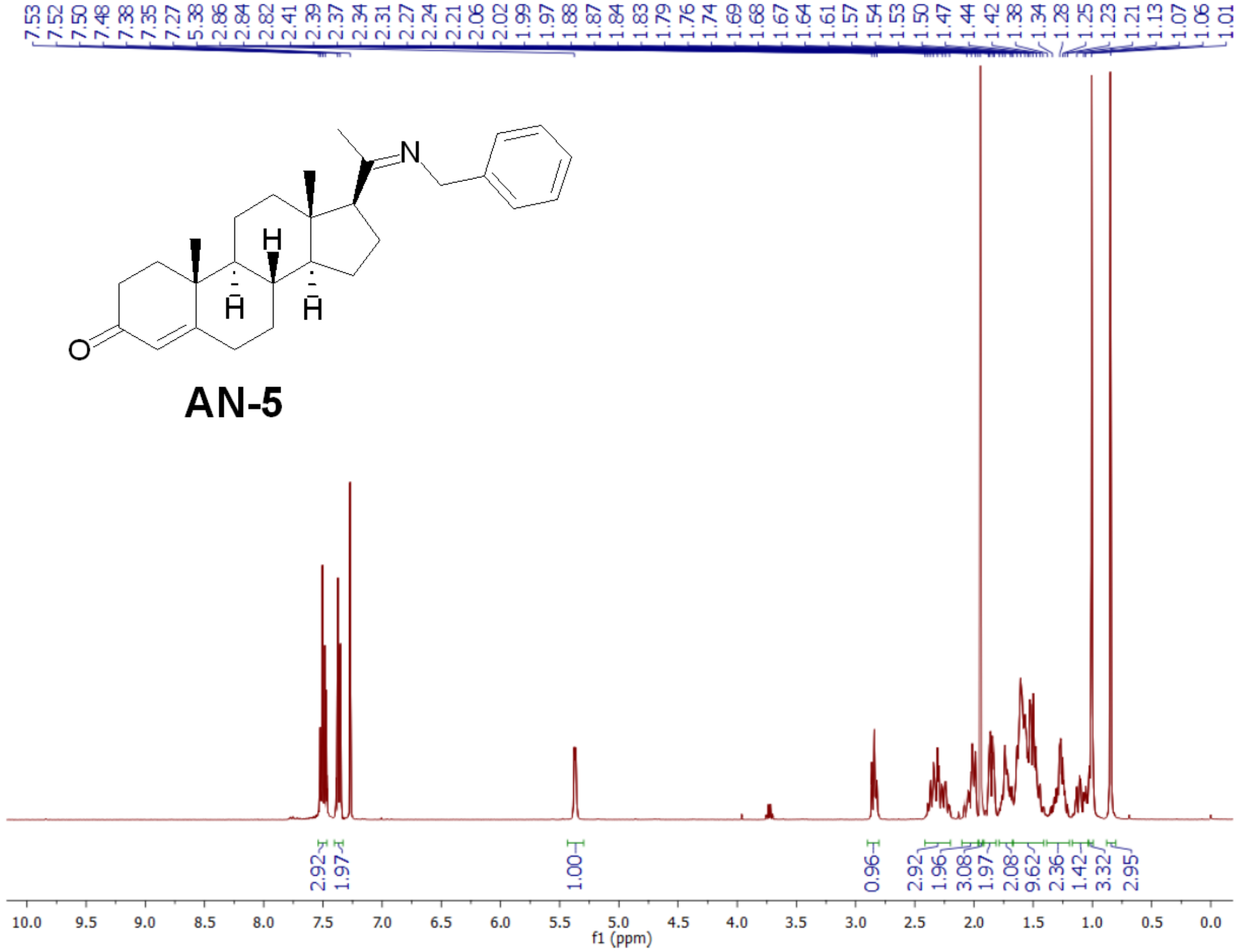

Figure S6: ¹H NMR spectrum of AN5 compound.

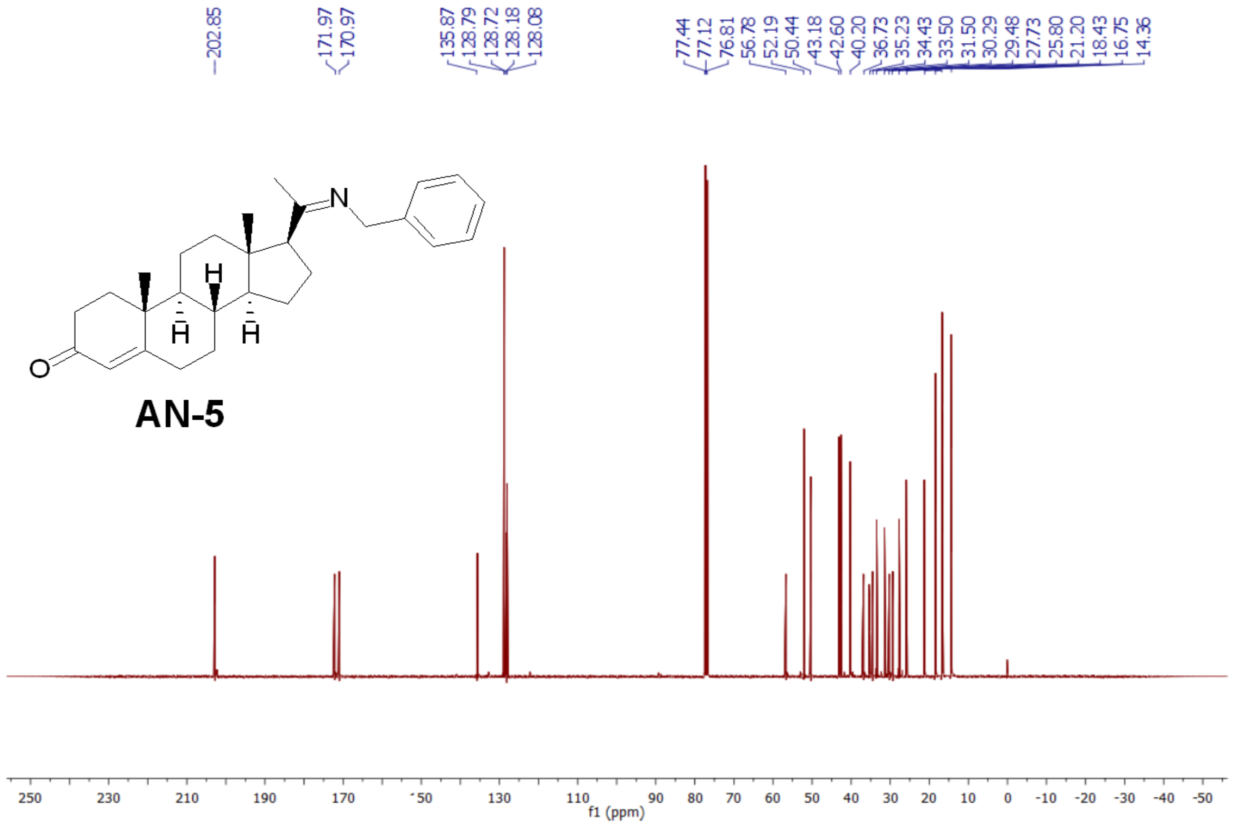

Figure S7: ^{13}C NMR spectrum of AN5 compound.
